# PROTOCOL: Impact of financial inclusion in low‐ and middle‐income countries: a systematic review of reviews

**DOI:** 10.1002/CL2.211

**Published:** 2018-04-18

**Authors:** Maren Duvendack, Philip Mader

## Background (see MECIR checklist, items 1 and 3)

### The problem, condition or issue

Financial inclusion is presently one of the most widely recognised areas of activity in international development. Financial inclusion initiatives have built upon donors’ experience with microfinance, but have displaced and superseded microfinance interventions in recent years with a more encompassing agenda of financial services for poverty alleviation and development (Mader 2016). With financial inclusion, policymakers and donors hope that access to financial services (including credit, savings, insurance and money transfers) provided by a variety of financial service providers (FSPs), of which microfinance institutions are a subset, will allow poor and low‐income households in low‐ and middle income countries to enhance their welfare, grasp opportunities, mitigate shocks, and consequently escape poverty, as well as advance macroeconomic development which is also expected to benefit poor/low‐income households. More recently, some donors have suggested behavioural changes (such as household spending decisions) to also be desired outcomes of access to financial services. Unlike most previous systematic reviews, which focused on microfinance interventions (or sub‐sets thereof), we explicitly adopt a broader scope to review any available systematic review or meta‐analysis evidence on financial inclusion as a whole field.

Systematic reviews, meta‐analyses and research syntheses (in short: meta‐studies) have sought to clarify the impacts from financial inclusion on poor people in low‐ and middle‐income countries, based on an array of different underlying studies which include quantitative and qualitative work based on long‐term and short‐term data. The bulk of these meta‐studies have been focused on microfinance, and many specifically on microcredit. The very different quality and approaches of these meta‐studies, and of the studies underlying them, however, pose a major challenge for policymakers, programme managers and practitioners in assessing the benefits and drawbacks of finance‐based approaches to poverty alleviation. Increasingly there is confusion about the impacts, and a risk of “cherry picking” among different findings. Further, many meta‐studies are not taking into account what is missing from their primary studies, which would affect understanding of the evidence, for example by not analysing or reporting gendered impacts. More recently, primary studies^1^ have also sought to understand the impacts of financial inclusion initiatives more broadly (Cull, Ehrbeck and Holle 2014; Demirgüc‐Kunt and Klapper 2013), but the systematic review evidence has not yet progressed as far as for microfinance.

Our primary aim is to gain better clarity about the impacts from financial inclusion on the poor by systematically reviewing the existing systematic reviews and meta‐analyses (in the broader field of meta‐studies). This generation of greater clarity through greater evidence systematisation is urgent given the strong focus on expanding access to financial services in the Sustainable Development Goals (SDGs), in particular SDG 1 on eradicating poverty^2^ and SDG 5 on achieving gender equality and women's empowerment^3^, and in light of the risks that some forms of financial inclusion may pose to vulnerable populations (Guérin et al. 2014). We have three sub‐objectives:

- to better inform the decisions of development donors, policymakers and programme managers by establishing what is known and not known about the impacts, using a meta review methodology;
- to facilitate better research by assessing the strengths and weaknesses of existing systematic reviews and meta‐analyses, and suggesting pathways toward improved and common standards and methods, and particularly more explicit attention to gendered equity determinants and a better use of qualitative studies;
- to understand the political economy of knowledge, which may explain which questions are asked and why, what analysis used and why, and how results are interpreted.

### The intervention

The field of financial inclusion in low‐ and middle‐income countries is diverse and complex, encompassing microfinance as the best‐known intervention in this space, but increasingly extending beyond it. Microfinance refers to the provision of financial services including loans, savings accounts, insurance (e.g. health, crop, life, credit life or default insurance), and money transfer services, specifically to poor and low‐income people in low‐ and middle income countries around the world who are not usually served by the regular banking sector, by *dedicated providers* who collectively identify as micro‐finance institutions (MFIs); these providers may range in size and type from small, local non‐profit NGOs to large commercial microfinance companies. Financial inclusion encompasses this set of services, while acknowledging a wider possible range of service providers, including community finance organisations, government programmes, commercial banks, fintech enterprises, and mobile network operators. It also highlights the connection of financial access with other services, for instance digital technologies, access to government welfare provision, mhealth services, or agricultural innovations. In short: microfinance is focused on specific financial services provided by a particular set of providers; financial inclusion more broadly aims to address poor and low‐income people's ability to access and use financial services for broader ends, and is agnostic about who provides them.

The most commonly‐provided services within financial inclusion still are microcredit loans, made to about 211 million families worldwide (Microcredit Summit Campaign 2015), with durations of around 12 months, which are repaid in weekly (and sometimes bi‐weekly or monthly) instalments, and are often guaranteed by group membership, small collateral, or personal guarantors. Savings and insurance services are usually offered only in conjunction with loans, but also sometimes independently. Money transfers and mobile payments services (i.e. financial technologies, or fintech, that have the potential to disrupt established business models of the inclusive financial space by delivering financial services via digital platforms) are a relatively new area of activity, which is still under development in many countries, but has achieved scale in parts of East Africa and South Asia; reviews of mhealth interventions will also be included to understand the role of mobile technologies in potentially shaping the provision of inclusive financial services. The space of financial inclusion is changing rapidly, and the purpose of this systematic review of reviews^4^ is to assess evidence for the broader range of inclusive financial services increasingly being offered, including but going beyond (micro)credit.

### How the intervention might work

The policy rationale behind financial inclusion activities is that the usage of financial services is expected to improve the lives of poor and low‐income people in low‐ or middle‐income countries (i.e. generate a positive impact) through one or more of three possible channels: (1) personal/household financial improvements, in the form of increased incomes, lower costs, building assets, sustainably consuming more goods and services, or managing the impacts of shocks better; (2) empowerment gains, primarily an increase in well‐being and health, and enhanced women's freedom, status, and recognition; (3) and macroeconomic development, when broader financial access drives inclusive growth or generates reductions in inequality. These channels and their most important impact pathways are shown schematically in the figure below.

**Figure 1:**
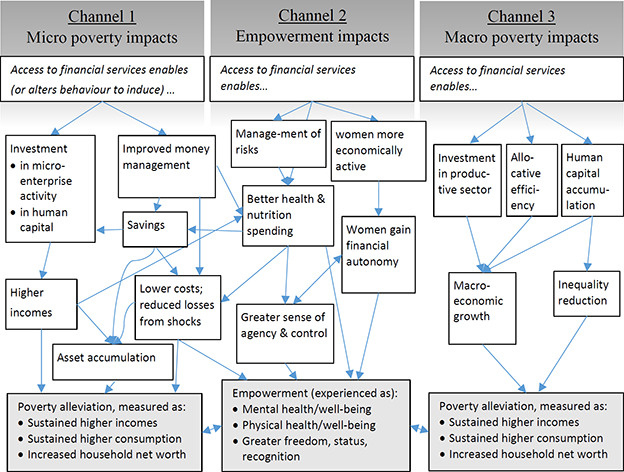
Pathways to financial inclusion impacts

Channel 1 refers to the (presumed) ability of individuals to translate financial services usage into financial improvements for themselves or their families. These improvements may result, to simplify, through two pathways: firstly, investments by the “economically active poor” (Ledgerwood 1999: 1) in revenue‐generating assets, primarily microenterprise (Grosh and Somolekae 1996), but also in human capital; secondly, through improved money‐management capability (ability to move money over space and time, from where/when it is earned to where/when it is needed) to enable smoothing consumption, saving, hedging against risk, and access to liquidity (Collins et al. 2007). Included in these channel is the potential of particular modes of financial service provision leading to changed attitudes and behaviours, wherein people reprioritise expenditures in ways that are more conducive poverty‐alleviation (e.g. less spending on “temptation goods”, more on asset accumulation), suggestions of which have grown more prominent in recent years (Banerjee et al. 2015; World Bank 2015).

Channel 2 refers to the (presumed) translation of financial services into the (subjectively‐experienced and/or objectively observable) empowerment of individuals who use them. The literature generally articulates two forms of empowerment: improved subjective well‐being and physical health regardless of gender, and empowerment for women. Physical outcomes may result from financial options which allow control of risk (e.g. insurance) or better spending on health (Dercon et al. 2012). Mental health/well‐being outcomes may result from improved life satisfaction, sense of self‐worth, feeling of being included and having more choices thanks to gaining access to financial services (Angelucci et al. 2014). Women's empowerment may result from access to financial services increasing women's status, freedom, and recognition (both within and outside the household), for instance when women are enabled to be more active in the public sphere, or increase their intra‐household economic decision‐making power, or gain greater autonomy (Kabeer 2005; Suri and Jack 2016).

Channel 3 refers to the potential for inclusive financial sectors to be conducive to macroeconomic development in low‐ or middle‐income countries, from which poor and low‐income people in turn benefit (Cull, Demirgüç‐Kunt, and Morduch 2013; World Bank 2014). The economic literature sees inclusive financial sector development as driving economic growth by mobilising savings and investments in the productive sector, and reducing information, contracting and transaction costs across the economy, leading to efficiency gains (Banerjee and Newman 1993; Galor and Zeira 1993; King and Levine 1993); poverty alleviation will result if poor people benefit from subsequent economic growth. Inclusive financial sector development is also seen as potentially reducing economic inequality, either indirectly (through growth leading to lower inequality) or through enabling lower‐income individuals to invest in their own human capital (Jalilian and Kirkpatrick 2005; Beck et al. 2007).

Notably, these channels of potential (that is: widely‐discussed, but not yet clearly demonstrated) impact of financial services usage on poverty (as well as their respective pathways) are interdependent, as indicated by some of the cross‐connections in the figure. But most existing reviews have focussed on individual channels or only certain pathways within them. A higher level of review systematisation will enable us to better understand the interconnections and contingencies between the different impact channels and pathways.

In each impact channel, furthermore, the possibility of adverse impacts (on average, or for parts of the population) must be considered, as there is no reason to assume the impacts will be positive. Among the adverse impacts that have been discussed at length in the literature so far are worsened impoverishment (Mosley 2001), financial and emotional stress (Ashta et al. 2015), debt traps and permanent indebtedness (Schicks 2010; Guérin et al. 2014), gender‐based violence and women's disempowerment (Rahman 1999), undermined economic development and social greater inequality (Bateman 2010; Sandberg 2012).

### Why it is important to do the review

While a large number of methodologically robust studies have systematically synthesised evidence on microfinance, the same cannot yet be said for financial inclusion more broadly. Some donor agencies, especially the World Bank, have carried out primary studies on financial inclusion of various types including microfinance facility to justify why financial inclusion policy matters, how it matters, and what it means to policymaking (cf. Cull, Ehrbeck and Holle 2014; Demirgüc‐Kunt and Klapper 2013; Demirgüc‐Kunt, Klapper and Singer 2017; World Bank 2014). But the existing research syntheses on financial inclusion (beyond microfinance) have been unsystematic in their approach.

Polanin et al (2017) provide 4 reasons for why systematic reviews of reviews are important:

1. They can contribute to the knowledge base going beyond what systematic reviews and meta‐analyses report examining trends over time and thus be particularly useful to policymakers, practitioners and researchers.
2. Where many systematic reviews on a given topic exist reporting discordant views, systematic reviews of reviews can be particularly useful to make sense of these diverging conclusions by comparing and contrasting the results of multiple systematic reviews.
3. They have the potential to conduct network meta‐analysis (Ioannidis, 2009) to allow comparisons of multiple treatment and control groups.
4. They can point out when systematic reviews need updating again.

Finally, it is worth noting that systematic reviews of reviews also have a role to play in translating knowledge into policy impact.

In the context of financial inclusion, without robust evidence that financial services generate significant and meaningful – ideally: transformative – impacts in poor people's lives, financial inclusion efforts would lack a clear justification in developmental or social policy terms. This can be said without pre‐judging the evidence. However, the existing systematic reviews and meta‐analyses which we are presently aware of (and which have focused on microfinance rather than financial inclusion broadly‐defined) have generated few strong or unambiguous results, suggesting that the improvements in poor people's lives that accrue from financial inclusion are relatively small or manifest mainly as intermediary impacts – changes in behaviours and spending patterns, rather than changes in incomes or well‐being –, at least in the shorter term. Presently, too little is known across different meta‐studies with different approaches, and we expect a systematic review of reviews will generate a clearer picture.

Existing systematic reviews and meta‐analyses have reviewed primary studies of many different types of financial services. As indicated in the table below, a substantial number of systematic reviews, meta‐analyses and research syntheses on financial inclusion and closely‐connected topics exist. However, the focus of the bulk of studies (in keeping with the activity focus of the financial inclusion sector) has been on credit and credit‐type (e.g. leasing) services, particularly those provided by MFIs. The evidence base on other services is smaller but growing rapidly, particularly in the area of mobile service provision and fintech for development.

The existing systematic reviews and meta‐analyses have followed diverse approaches. Some of the systematic reviews are fairly broad, aiming to cover the whole microfinance spectrum (e.g. Duvendack et al. 2011). Others cover specific interventions, such as microcredit (e.g. Vaessen et al. 2014), formal banking services (Pande et al. 2012), microenterprise (e.g. Grimm and Paffhausen 2015), microsavings and microleasing (Stewart et al. 2012), and microinsurance (Cole et al. 2012). Some systematic reviews focus on particular populations, such as Sub‐Saharan African recipients (e.g. Stewart et al. 2010), particular methods of providing financial services, such as self‐help groups (e.g. Brody et al. 2016) or particular outcomes, such as health (e.g. Leatherman et al. 2012) or empowerment (Vaessen et al. 2014; Brody et al. 2016). The systematic reviews also differ by focus, many covering effectiveness evidence, but others incorporating participant views (e.g. Brody et al. 2016) and barriers or enablers of uptake and effectiveness (e.g. Panda et al. 2016) including innovations in information and communications technology (e.g. Gurman et al. 2012, Jennings and Gagliardi 2013, Sondaal et al. 2015, Lee et al. 2016).

The systematic reviews and meta‐analyses use a range of methodologies to synthesise the evidence, including theory‐based approaches, narrative syntheses and statistical meta‐analyses. Many of them have not been conducted to standards that would support a ‘high confidence’ rating^5^; not all meta‐studies that have impacted policy discussions have used a systematic methodology (Odell 2010; Bauchet et al. 2011; Beck 2015). In addition, the majority of systematic reviews and meta‐analyses are available in technical reports where there is no transparent decision rule for determining implications of the findings, including critical appraisal and strength of evidence tools like GRADE assessment (Guyatt et al. 2013) and user‐friendly presentation of results (e.g. translating standardised effect sizes into metrics commonly used by decision makers). In addition, there is no overall synthesis of the implications for policy, programming, practice and research for the sector from this body of synthesised evidence.

Below is a chronological overview of many known systematic reviews, meta‐analyses and research syntheses of financial inclusion interventions:

This array of meta‐studies, often focused on different geographies and dealing with different interventions, presents policymakers and practitioners with a potentially perplexing mosaic of findings, non‐findings, and knowledge gaps. Our systematic review of reviews will bring a systematic overview about what is known about what aspects of financial inclusion (what, where, how?) and which gaps and white spaces remain. To visualise these gaps and white spaces, we will adopt a mapping approach which is a loose adaptation of the more formal evidence gap map approach developed by Snilstveit et al (2013) and Gough et al (2013). Our map will be guided by a range of parameters, e.g. intervention type, outcome measures, geographical focus, etc. which in turn will inform our synthesis approach which, among other things, will also focus on the following unresolved questions (discussed in more depth in the section outlining our approach to data synthesis):

- What can explain which questions are asked in some systematic reviews and meta‐studies about the impact of financial inclusion, and which ones not?
- What can explain different interpretations of results from existing studies?

A clear mapping of knowledge gaps will allow policy‐related research funders to better direct research funds towards addressing the gaps, and the systematic reviewing of known impacts will allow policymakers to focus their efforts on those interventions that are known to work best, on where they work best, or otherwise to eschew or improve them. Our stakeholder engagement strategy will include a policy brief, a collection of blogs, and dissemination events, as well as working with our advisory board to disseminate the findings.

## Objectives (see MECIR checklist, item 2)

The objective of this systematic review of reviews is to systematically collect and appraise the existing systematic reviews and meta‐analyses of financial inclusion impacts, analyse the strength of the methods used, synthesise the findings from those systematic reviews and meta‐analyses, and report implications for policy, programming, practice and further research.

Systematic reviews of reviews are undertaken in other sectors for which evidence is widely available, especially health (Becker and Oxman 2008) and recently education (Polanin et al. 2017), but they are non‐existent in international development, and thus this study will address a notable gap.^6^ It provides the opportunity to develop and pilot an evidence synthesis approach in a sector where there is a large body of evidence of variable quality, but a systematic appraisal and synthesis of the body of systematic reviews and meta‐analyses is lacking. Polanin et al (2017) provide useful guidance on how best to conduct such systematic reviews of reviews; they point towards methodological challenges of such reviews and suggest ways forward to improving them.

This study will critically review existing approaches to systematic reviews of reviews with a view to further developing systematic review of review methods and it will aim to answer the following questions to gain better clarity about financial inclusion impacts:

- Impacts:
  ∘ What is known from existing systematic reviews and meta‐analyses about the poverty impacts (social, economic, and behavioural) of different types of inclusive financial services (e.g. credit, savings, insurance, money transfers), regardless of provider, on poor and low‐income people in low‐ and middle income countries?^7^ This includes the poverty impacts from macroeconomic development to the extent that it results from financial inclusion.^8^
  ∘ What is known from existing systematic reviews and meta‐analyses about the gendered differential impacts of different types of financial inclusion activity (e.g. credit, savings, insurance, money transfers) – in other words, what does the evidence tell us about how gendered social and structural determinants of inequality affect intervention effects, as well as whether or not financial services empower women, and in what ways, in low‐ and middle income countries?
  ∘ What is known from existing systematic reviews and meta‐analyses about reasons for financial services uptake, or other participant views about the financial services on offer?
- Methodology:
  ∘ Including using a gender and equity lens, what methods and standards have the systematic reviews and meta‐analyses used to draw conclusions from the studies they reviewed?
  ∘ What difference does the choice of methods and standards make to the results?
  ∘ How could the methods and standards be improved in order to draw more robust and reliable conclusions via systematic reviews and meta‐analyses?

## Methodology

### Criteria for including and excluding studies (see MECIR checklist, items 5‐14)

#### Types of reviews (see MECIR checklist, item 9)

We will include studies which self‐identify as systematic reviews and or meta‐analyses of the impacts of financial inclusion (including, but not limited to, microfinance). These in turn will have focused on synthesising quantitative, qualitative and or mixed methods evidence. According to the Campbell Collaboration,

“A systematic review summarizes the best available evidence on a specific question using transparent procedures to locate, evaluate, and integrate the findings of relevant research” (The Campbell Collaboration 2014, p.6).

In the Cochrane Handbook (Higgins and Green 2011), the following definition of systematic reviews is outlined which we will adopt:

“A systematic review attempts to collate all empirical evidence that fits pre‐specified eligibility criteria in order to answer a specific research question. It uses explicit, systematic methods that are selected with a view to minimizing bias, thus providing more reliable findings from which conclusions can be drawn and decisions made” (Section 1.2 in Higgins and Green 2011).

Higgins and Green (2011) specify the key elements a systematic review should contain:

- A set of clearly stated objectives and pre‐defined eligibility criteria
- A methodology that is clearly defined allowing reproducibility
- A search strategy that allows the identification of studies meeting the pre‐defined eligibility criteria
- A critical appraisal of included studies
- A systematic synthesis, in many cases systematic reviews adopt a meta‐analytical approach which is a statistical method to synthesise the results of primary studies included in a systematic review

To identify meta‐analyses, we adopt the definition of the Cochrane Handbook (Higgins and Green, 2011):

“Meta‐analysis [is] the statistical combination of results from two or more separate studies” to produce an overall statistic with the aim to provide a precise estimate of the effects of an intervention (Section 9.1.2 in Higgins and Green 2011).

It should be noted that not every systematic review automatically contains a meta‐analysis, e.g. if primary studies are too heterogeneous in terms of study designs, conceptual framings and or outcomes, then a meta‐analysis may not be appropriate. Furthermore, occasionally meta‐analyses are published separately without drawing on the broader systematic review they may have been originated from.

We exclude any evidence that did not meet the definitions we outlined above.

#### Types of participants (see MECIR checklist, item 5)

The scope of the systematic reviews and meta‐analyses we will include may be diverse (different questions are often addressed; a range of linked interventions are examined such as credit, savings, insurance, leasing, money transfers etc.) but there is considerable overlap in terms of their population of interest. Almost all systematic reviews and meta‐analyses focus on the impacts of financial inclusion on poor households based in low‐ or middle‐income countries (using the World Bank definition^9^). In other words, our population is the population of participants in inclusive finance activities that are conducted in low‐ and middle‐income countries. Where systematic reviews and meta‐analyses include evidence from high‐income countries, we will only consider the findings that are presented for low‐ and middle‐income countries; we will also consider systematic reviews and meta‐analyses covering particular regions within low‐ and middle income countries, e.g. Sub‐Saharan Africa or fragile and conflict‐affected areas.

At the primary study level, our population of interest would be participants taking part in inclusive finance activities in low‐ and middle‐income countries.

#### Types of interventions (see MECIR checklist, item 7)

In this systematic review of reviews, we will include all systematic reviews and meta‐analyses that address at least one or more types of intervention for financial inclusion, as described above. In the majority, we expect the interventions will be one or more sub‐categories of microfinance: microcredit, micro‐savings, micro‐insurance, micro‐leasing, and/or money transfers. However, our search strategy explicitly targets the broader range of inclusive finance activities, such as mobile monies, index insurance, or savings promotion. For our purposes, to warrant inclusion of the systematic review or meta‐analysis, the reviewed intervention must have at least one financial service as an essential element of the intervention – for instance, not all systematic reviews of mhealth interventions would qualify for inclusion, but systematic reviews of mhealth interventions that require participants to purchase an insurance service would. The key is that the intervention is fundamentally a financial service directed at poor and low‐income people.

At the primary study level, our intervention of interest would be interventions that address at least one or more types of financial inclusion interventions.

#### Types of outcome measures (see MECIR checklist, items 8 and 14)

Existing systematic reviews and meta‐analyses of financial inclusion typically examine a wide range of poverty indicators (including income, assets, expenditure, personal networks, gender/empowerment, well‐being, health, etc.). In this systematic review of reviews, we will include all systematic reviews and meta‐analyses that address at least one or more of these domains. We will group the indicators in three categories of impacts: social, economic, or behavioural. We will not distinguish between primary or secondary outcomes but consider all outcome measures.

Our systematic review of reviews will also assess the evidence for outcomes further back along the causal chain; most importantly rates of uptake, and then investment in productive activity, human capital accumulation, improved money management, savings accumulation, risk/shock management, health and nutrition spending, and women's economic activity. These might be enablers of improvements on poverty indicators (over a longer term) even if, importantly, should not in themselves be taken as evidence of impact in terms of poverty alleviation.

At the primary study level, our outcomes of interest would be outcomes that address at least one or more of the poverty domains described above.

#### Timeframe

The first systematic reviews engaging with financial inclusion issues (Stewart et al 2010, Duvendack et al 2011) indicated that no systematic reviews existed prior to their reviews. The primary studies these two systematic reviews included date back to the late 1990s reporting on data that was collected in the early 1990s – this coincides with rigorous impact evaluations of financial inclusion becoming more mainstream, hence our searches will be limited to 2010 onwards.

#### Language

No restriction was placed on language of papers.

We do not expect to make any changes to the eligibility criteria set out in this section but should we need to make any adjustments we will justify and documents these carefully and any changes will be in line with the objectives of this systematic review of reviews *(relates to MECIR checklist, item 13)*.

Evidence will be included irrespective of its publication status (*relates to MECIR checklist, item 12)*.

### Search strategy (see MECIR checklist, items 19, 24, 32, 33, 35, 36 and 37)

We will adopt a multi‐pronged search strategy which was informed by Kugley et al (2016) and that explores bibliographic databases to identify published literature, institutional websites for published and unpublished literature, and back‐referencing from recent systematic reviews (see Table 1 above) to ensure additional sources are identified.

**Table 1:** Overview of systematic reviews, meta‐analyses and research syntheses of financial inclusion interventions

**Authors**	**Details**	**Geographical focus**	**Funder**
*Odell, 2010*	Research synthesis	Worldwide	Grameen Foundation
*Stewart et al, 2010*	SR; quantitative evidence only	Sub‐Saharan Africa	DFID
*Duvendack et al, 2011*	SR; quantitative evidence only	Worldwide	DFID
*Bauchet, et al, 2011*	RCT evidence only – not a SR	Worldwide	CGAP
*Leatherman et al, 2012*	SR; microfinance and health	Worldwide	University of North Carolina
*Pande et al, 2012*	SR; formal banking services	Worldwide	DFID
*Stewart et al, 2012*	SR; includes micro‐leasing, quantitative evidence only	Worldwide	DFID
*Grimm and Paffhausen, 2015*	SR; micro‐entrepreneurs	Worldwide	KfW Group
*Maitrot and Niño‐Zarazúa, 2013*	SR; quantitative evidence only	Worldwide	Unclear
*Cole et al, 2012*	SR; micro‐insurance focus, quantitative only	Worldwide	DFID
*Gurman et al, 2012*	SR; mhealth	Worldwide	Unclear
*Yang and Stanley, 2013*	Meta‐analysis only, focus on income	Worldwide	Self‐funded
*Jennings and Gagliardi, 2013*	SR; mhealth and gender focus	South Asia, Sub‐Saharan Africa	Unclear
*Vaessen et al, 2014*	SR including meta‐analysis; empowerment focus	Worldwide	3ie
*Awaworyi, 2014*	Meta‐analysis only	Worldwide	Self‐funded
*Arrivillaga and Salcedo, 2014*	SR; focus on HIV/AIDS prevention	Worldwide	Unclear
*Aranda‐Jan et al, 2014*	SR; mhealth	Africa	Unclear
*Madhani, Tompkins*, *Jack and Fisher, 2015*	Modified SR; focus on women's mental health	Worldwide	Unclear
*Beck, 2015*	Research synthesis	Worldwide	World Bank
*Sondaal et al, 2015*	SR; mhealth	Worldwide	Authors received no specific funding
*Devi et al, 2015*	Updated SR; mhealth	Sub‐Saharan Africa	Unclear
*Watterson et al, 2015*	SR; mhealth	South Asia, Sub‐Saharan Africa	Unclear
*Agarwal et al, 2015*	SR; mhealth	South Asia, Sub‐Saharan Africa, Latin America & Carribean	mPowering Frontline Health Workers – USAID & Johns Hopkins University
*Brody et al, 2016*	SR; SHGs/women's empowerment	Worldwide	3ie
*Gopalaswamy et al, 2016*	SR; quantitative evidence only	South Asia	DFID
*Panda et al, 2016*	SR, health financing, insurance	Worldwide	3ie
*Lee et al, 2016*	SR and meta‐analysis, mhealth	Worldwide	WHO
*White et al, 2016*	SR; mhealth	South Asia	National Institutes of Health
*Amoakoh‐Coleman et al, 2016*	SR; mhealth	Middle East & North Africa, Sub‐Saharan Africa	Netherlands Organization for Scientific Research (NWO)
*Steinert et al, forthcoming*	SR; micro‐savings	Sub‐Saharan Africa	Unclear

We will search the following bibliographic databases:

- Business Source Premier (Ebsco)
- Academic Search Complete (Ebsco)
- Econlit – Via Ebsco Discovery Service
- Repec – Via Ebsco Discovery Service
- World Bank e‐Library – Via Ebsco Discovery Service
- Scopus (Elsevier)
- Web of Science

The following institutional websites will be searched:

*Financial inclusion‐specific institutions and web portals:*

- CGAP: www.cgap.org
- Microbanking Bulletin: www.themix.org
- Microfinance Gateway: www.microfinancegateway.org
- Microfinance Network: www.mfnetwork.org
- SEEP: http://www.seepnetwork.org
- Grameen Foundation
- BRAC Research and Evaluation Division
- Alliance for Financial Inclusion
- Accion Center for Financial Inclusion

*Multilateral and bilateral and non‐governmental donor organizations:*

- World Bank (WB e‐library was searched within Ebsco's Discovery Service but will also be searched and screened online via the World Bank's website)
- African Development Bank
- Asian Development Bank
- Inter‐American Development Bank
- DFID – R4D website
- USAID

*Research institutions and research networks:*

- Center for Global Development
- J‐PAL
- 3ie databases on systematic reviews
- ELDIS
- SSRN
- ResearchGate
- Academia.edu

After completing the screening process, we will also run citation searches on included systematic reviews and meta‐analyses in Google Scholar, Scopus and Web of Science to identify more recent systematic reviews or meta‐analyses not retrieved in database searches.

We piloted our key search terms (see Appendix 1 for full search strategies) and ran preliminary searches in Econlit (Ebsco) (510 hits), Scopus (1035 hits), Repec (Ebsco) (238 hits), Academic Search Complete (Ebsco) (366 hits), and Web of Science (2014 hits). Search strategies were constructed using both textwords (title/abstracts) and where available index terms. Each strategy consisted of 3 parts – Intervention (financial inclusion, microfinance and other relevant terms), Study design (adapted from 3ie's search filter for its systematic review database), and LMICs (adapted from the Cochrane EPOC Group's LMICs filter based on World Bank definition of LMICs). We adjusted our search strategy for each database and web source. No restriction was placed on language of papers but all searches were limited to 2010 onwards (rationale provided above) and only English language papers were identified. We will adopt a snowballing, also called reference harvesting, approach to ensure we have not missed any key systematic reviews or meta‐analyses. We will also consult our advisory board to get their views on the sample of included studies. We will update our searches for all relevant databases within 12 months before publication of our study and screen all new systematic reviews and meta‐analyses using the eligibility criteria outlined above.

### Selection of studies (see MECIR checklist, items 39 and 41)

One review author (MD) will screen all titles and abstracts of the systematic reviews and meta‐analyses identified by the search. The second review author (PM) will independently review each systematic review and meta‐analysis for inclusion to confirm the inclusion decision of the first review author (MD). Full texts will be obtained and screened when a decision cannot not be made based on title and abstract screening. Disagreements will be resolved by discussion or by involving a third party (e.g. a member of the advisory board) if a consensus cannot be reached.

A PRISMA flow diagram will be used to summarise the study selection process and a table with the characteristics of excluded studies will be included in the appendix.

### Description of methods used in systematic reviews (see MECIR checklist, item 44)

The systematic reviews and meta‐analyses we will include will have included primary studies that employed quantitative, qualitative and mixed methods approaches. Hence, many of the systematic reviews and meta‐analyses in our study sample will have adopted a narrative synthesis approach to deal with the methodological diversity found in the included primary studies (e.g. Stewart et al 2010 and 2012, Duvendack et al 2011). In some cases, however, meta‐analysis is feasible and the preferred synthesis approach (e.g. Yang and Stanley 2013, Awaworyi 2014, Lee et al 2016). In very few cases, a combination of qualitative and quantitative synthesis approaches can be found (e.g. Vaessen et al 2014).

### Criteria for determination of independent reviews (see MECIR checklist, items 40 and 42)

Some of the systematic reviews and meta‐analyses in our study sample will have been published in multiple places, e.g. they may have been published as a Campbell systematic review but also as a peer‐reviewed journal article (e.g. Vaessen et al 2014). Or they may have been published on DFID's R4D website as well as a peer‐reviewed journal article (e.g. Stewart et al 2012). Where this is the case, we will treat them as duplicate reviews with data extracted from the most comprehensive review. Should we identify multiple versions of the same systematic review or meta‐analysis, we will only include the latest updated version. An issue that remains after removing duplicate systematic reviews and meta‐analyses is that of overlap. In our sample of included systematic reviews, we may find reviews that included some of the same primary studies. One way to address overlap is to present a matrix (see Polanin et al 2017) that includes all primary studies captured in the systematic reviews with a high and medium conference rating, this would allow us to understand the extent of overlap, i.e. which primary studies were included in which one of the high quality systematic reviews in our study sample.

Where systematic reviews or meta‐analyses do not report data in useable formats, we will still attempt to include them as long as they meet our eligibility criteria.

### Details of study coding categories (see MECIR checklist, items 43, 46, 47, 50 and 51)

Data will be extracted by a research assistant (RA) using an Excel spreadsheet and independently checked by the two review authors (MD, PM). In case of disagreements, they will be resolved by discussion. The original authors of included systematic reviews and meta‐analyses will be contacted where data are missing.

We will extract data on the following areas (for details see Table 2 below which was informed by Sniltsveit et al 2014):

1. Context
2. Type of intervention
3. Type of review, design and methods used
4. Outcome measures
5. Quality assessment
6. Study results and findings

**Table 2:** Data extraction form (template)

**Data extraction items**	Details
1.**Context**	Source Author Publication year Geographical focus (e.g. continent, countries, regions) Funding source
**2. Type of intervention**	Details of the population as discussed in the reviews (e.g. household, individual, enterprise; type of finance user, i.e. multiple borrower/saver, repeat borrower/saver; gender or other person characteristics, e.g. women focus or youth focus) Broad category – type of product/service offered, ensure intervention has at least one essential financial service element Detailed sub‐category of product (e.g. credit to existing businesses only, group savings account, etc.) Comparator, i.e. comparing against nothing at all or against the next best alternative Duration of intervention (e.g. length of exposure to intervention) Modality of intervention – group vs individual Location of intervention – urban/rural Focus on women only (yes/no)
**3. Type of review; design and methods**	Research question and review objectives – list actual question, plus clearly stated (yes/no) Inclusion criteria – clearly stated (yes/no) Search methods ‐ e.g. number of databases, dates of search provided, search strategy/key words provided, additional search methods reported, any search restrictions (by language, timeframe?) Study selection methods – clearly reported (yes/no), independent screening, full text review, consensus procedure for agreements Number of included studies Types of included studies Types of data extraction methods ‐ clearly reported (yes/no), independent screening Types of data synthesis approaches (quantitative/qualitative) Subgroup analysis conducted (yes/no) Discussion of publication bias (yes/no)
**4. Outcome measures**	Outcome definition, i.e. type of outcome measure to be grouped by social, economic, behavioural Unit of measurement (e.g. at household or individual level, composition of empowerment indices)
**5. Quality assessment**	Quality of review methods, their use and application – to be assessed using data extracted as part of ‘3. Type of review; design and methods’ which will feed into AMSTAR rating GRADE rating provided (yes/no) Name of other quality assessment tools and their quality scores Researcher bias/Conflict of interest
**6. Study results and findings**	For each outcome: ∘ Sample size ∘ Type of effect size ∘ Magnitude and direction of effect size if reported to allow a comparison across included studies

We will aim to extract the most detailed data (also numerical data if available) to allow similar analyses of included studies.

We will primarily be extracting information at the systematic review level. However, for systematic reviews classified as high and medium confidence we will also extract information at the primary study level on, e.g. especially individual programme design, quality, etc. We may also explore whether effect size estimates can be extracted from t‐statistics if feasible and necessary.

### Assessment of quality (see MECIR checklist, items 20, 52, 53, 54 and 61)

#### Assessment of methodological quality of included reviews (see MECIR checklist, items 52, 53 and 54)

The quality of the included systematic reviews and meta‐analyses will be assessed using the 3ie critical appraisal checklist^10^, which is a variation of the checklist developed by the Specialist Unit for Review Evidence (SURE) in 2013. The objective of the original SURE^11^ checklist was to allow a critical appraisal of systematic reviews to ensure that minimum levels of methodological rigour are met across included reviews. We will explore extensions to the 3ie checklist in collaboration with 3ie and may add a critical appraisal component that relates to the explicit use of theory in systematic reviews and to what extent an analysis of the causal chain has been undertaken.

Furthermore, to corroborate the findings of the 3ie critical appraisal checklist, we will also be employing the ‘A MeaSurement Tool to Assess systematic (AMSTAR 2) developed by Shea et al (2017) which is often used in the context of Cochrane overview studies. AMSTAR 2 is building on the original AMSTAR tool developed by Shea et al (2007), it has 16 criteria^12^ and each will be given a rating: ‘yes’, ‘partial yes’ or ‘no’ allowing the user to make a broad assessment of the quality of the included reviews.

The 3ie critical appraisal checklist and AMSTAR 2 tool will be applied independently by both review authors (MD, PM), disagreements will be resolved by discussion or by involving a third party (e.g. a member of the advisory board) if a consensus cannot be reached.

#### Assessment of the quality of the evidence in reviews (see MECIR checklist, items 61, 76 and 77)

We will extract GRADE ratings from each included systematic review to assess the quality of the evidence. It is highly likely that many of the included systematic reviews will have adopted quality assessment approaches other than GRADE, where this is the case we will report the tool that was used and record its overall quality score. We will re‐evaluate the quality score (i.e. potentially downgrade or upgrade it) retrospectively using the main GRADE criteria related to risk of bias, inconsistency, imprecision, indirectness and publication bias (Guyatt et al 2008, ^13^). This re‐evaluation will only be done for systematic reviews that have a medium or high confidence rating. The quality assessment will be conducted independently by both review authors (MD, PM), disagreements will be resolved by discussion or where necessary by involving a third party (i.e. a member of the advisory board).

### Data synthesis and presentation (see MECIR checklist, items 21, 78 and 79)

The included systematic reviews will have adopted a wide range of synthesis approaches ranging from narrative syntheses to meta‐analyses. Having reviewed the various synthesis methods set out by Barnett‐Page and Thomas (2009), we will adopt a narrative synthesis approach as this accommodates both quantitative and qualitative information and is thus best suited for our purpose.

#### Quantitative information

Some of the systematic reviews in our sample will have taken a meta‐analytical approach; where this is the case, we will explore the possibility of reporting the pooled effects sizes for all outcomes and/or explore whether there is value in compiling and synthesising the magnitudes of the effect sizes from their included primary studies. Where systematic reviews drawing on meta‐analytical techniques do not report this information, we will downgrade the review^14^. Where feasible, we will attempt to back‐translate effect size data (e.g., such as standardised mean differences) into a (from a policy perspective) more understandable magnitude of units (e.g. mean differences in standard units and or percentage changes).

#### Qualitative information

We expect that the majority of the included systematic reviews will have adopted qualitative synthesis approaches. To synthesise the qualitative evidence, we will use a thematic synthesis approach, which is particularly useful to understand systematic reviews (including effectiveness reviews) which address questions in relation to appropriateness and acceptability of interventions as well as to understanding barriers and enablers of an intervention. Thematic synthesis first organises content into ‘descriptive’ themes, which are then explored to generate ‘analytical’ themes for further analysis (Barnett‐Page and Thomas 2009).

In addition, we will present our findings according to the statistical information available in each systematic review, which may often be a textual commentary. This commentary will be enhanced by drawing on summary tables and figures using frequencies and percentages to describe and summarize the evidence we collected from the included reviews (see Smith et al 2011 for suggestions for summary tables). Where possible, we will also attempt to report findings in metrics of effect sizes and 95% confidence intervals, which may require the use of standard formulae to translate between effect sizes (e.g. see Sanchez‐Meca et al 2003 for guidance) – we should note that this is an exploratory exercise.

Baker et al (2014) argue that the emphasis of systematic reviews of reviews should be on the presentation of the results and conclusions of the included reviews in accordance with their overall objectives. With this in mind, we may organise our synthesis by data extraction areas (as outlined above):

1. Context
2. Type of intervention
3. Type of review, design and methods used
4. Outcome measures
5. Quality assessment
6. Study results and findings

Following Jadad et al (1997), the synthesis will also be guided by the following two questions introduced above as part of the rationale for doing the review:

- What can explain which questions are asked in some systematic reviews and meta‐studies about the impact of financial inclusion, and which ones not?
- What can explain different interpretations of results from existing studies?

The findings from our synthesis will inform the conclusions of this study; we will not stray beyond the studies included in this review when discussing the implications for research and practice.

### Treatment of qualitative research

A large number of the included systematic reviews will have adopted a qualitative synthesis approach; where this is the case, we will use appropriate data synthesis and presentation formats, as outlined in the previous section, to allow a comparison across systematic reviews.

### Sub‐group analysis (relates to MECIR checklist, item 22)

We will report sub‐group analyses adapting the PROGRESS‐Plus checklist^15^ which was originally developed for Cochrane reviews focusing on health equity. Sub‐group analyses enhance our understanding of impact heterogeneity, i.e. impacts of certain elements of financial inclusion interventions may differ by gender, ethnic background, poverty level, etc. Reporting sub‐group analyses may allow us to comprehend which interventions (or elements thereof) may or may not be effective in relation to certain sub‐groups in the population.

## Review authors

**Lead review author:** The lead author is the person who develops and co‐ordinates the review team, discusses and assigns roles for individual members of the review team, liaises with the editorial base and takes responsibility for the on‐going updates of the review.

**Table:** 

Name: Maren Duvendack	
Title: Dr.	
Affiliation:	University of East Anglia
Address:	School of International Development Norwich Research Park
City, State, Province or County:	Norwich
Postal Code:	NR2 3HT
Country:	United Kingdom
Phone:	07788777818
Email:	m.duvendack@uea.ac.uk
**Co‐author(s):** (There should be at least one co‐author)	
Name: Philip Mader	
Title: Dr.	
Affiliation:	Institute of Development Studies
Address:	Library Road, University of Sussex
City, State, Province or County:	Brighton, East Sussex
Postal Code:	BN19RE
Country:	United Kingdom
Phone:	07711 761418
Email:	p.mader@ids.ac.uk

*Duplicate the co‐author table as necessary to include all co‐authors*.

## Roles and responsibilities

- Content: Maren Duvendack, Philip Mader
- Systematic review methods: Maren Duvendack, John Eyers (search strategy)
- Statistical analysis: Maren Duvendack, Philip Mader
- Information retrieval: John Eyers, Ada Sonnenfeld

## Sources of support

The International Initiative for Impact Evaluation (3ie) is funding this work.

## Declarations of interest

Maren Duvendack was lead author on one systematic review (Duvendack et al. 2011) and contributing author on one (Vaessen et al. 2014).

Philip Mader conducted an overview of (only most recent) financial inclusion impact evidence in early 2017 for a consultancy (unpublished).

## Preliminary timeframe

30 July 2018

## Plans for updating the review

Ideally, this review should be updated every five years to include new systematic reviews on financial inclusion. However, regular updates are subject to availability of funding.

## AUTHOR DECLARATION

### Authors’ responsibilities

By completing this form, you accept responsibility for preparing, maintaining and updating the review in accordance with Campbell Collaboration policy. The Campbell Collaboration will provide as much support as possible to assist with the preparation of the review.

A draft review must be submitted to the relevant Coordinating Group within two years of protocol publication. If drafts are not submitted before the agreed deadlines, or if we are unable to contact you for an extended period, the relevant Coordinating Group has the right to de‐register the title or transfer the title to alternative authors. The Coordinating Group also has the right to de‐register or transfer the title if it does not meet the standards of the Coordinating Group and/or the Campbell Collaboration.

You accept responsibility for maintaining the review in light of new evidence, comments and criticisms, and other developments, and updating the review at least once every five years, or, if requested, transferring responsibility for maintaining the review to others as agreed with the Coordinating Group.

### Publication in the Campbell Library

The support of the Coordinating Group in preparing your review is conditional upon your agreement to publish the protocol, finished review, and subsequent updates in the Campbell Library. The Campbell Collaboration places no restrictions on publication of the findings of a Campbell systematic review in a more abbreviated form as a journal article either before or after the publication of the monograph version in Campbell Systematic Reviews. Some journals, however, have restrictions that preclude publication of findings that have been, or will be, reported elsewhere and authors considering publication in such a journal should be aware of possible conflict with publication of the monograph version in Campbell Systematic Reviews. Publication in a journal after publication or in press status in Campbell Systematic Reviews should acknowledge the Campbell version and include a citation to it. Note that systematic reviews published in Campbell Systematic Reviews and co‐registered with the Cochrane Collaboration may have additional requirements or restrictions for co‐publication. Review authors accept responsibility for meeting any co‐publication requirements.

**I understand the commitment required to undertake a Campbell review, and agree to publish in the Campbell Library. Signed on behalf of the authors**:

**Form completed by: Maren Duvendack, Philip Mader**

**Date: 15 December 2017**

## Footnotes

1 We use the term primary studies to denote individual studies that make up a systematic review, meta‐analysis or research synthesis.

2
https://sustainabledevelopment.un.org/sdg1

3
https://sustainabledevelopment.un.org/sdg5

4 We use the term systematic review of reviews to denote our population of studies consisting of systematic reviews and meta‐analyses.

5 E.g. 3ie uses an assessment tool in providing confidence ratings for all systematic reviews they include in the 3ie systematic reviews repository. The assessment tool is available at: http://www.3ieimpact.org/media/filer_public/2012/05/07/quality_appraisal_checklist_srdatabase.pdf.

6 Evans and Popova (2015) produced a review that claimed to find divergent findings in six “systematic reviews” of education programmes. However, the authors did not screen or critically appraise included reviews according to standard definitions. Hence, further analysis indicated only one of the included studies was undertaken using systematic review methods, the other five being literature reviews and meta‐analyses which did not use comprehensive approaches to select, appraise and/or synthesise the evidence (Snilstveit, Vojtkova and Phillips, 2015).

7 Our review will disaggregate the impacts of different services provided by different provider‐types, and examine heterogeneous impacts on different user groups, as much as the data permits.

8 We are not presently aware of any systematic reviews or meta‐analyses that address this type of impact, but hope to find and include such evidence to the extent that it matches our inclusion criteria.

9 The World Bank definition of lower/middle income countries is used: https://datahelpdesk.worldbank.org/knowledgebase/articles/906519‐world‐bank‐country‐and‐lending‐groups

10
http://www.3ieimpact.org/media/filer_public/2012/05/07/quality_appraisal_checklist_srdatabase.pdf

11
https://www.cardiff.ac.uk/__data/assets/pdf_file/0005/212756/SURE_SR‐Checklist_2015.pdf

12 See online checklist for details: https://amstar.ca/Amstar_Checklist.php

13 A good overview of all GRADE related papers can be found here: http://bestpractice.bmj.com/info/toolkit/learn‐ebm/what‐is‐grade/

14 We explored the possibility of conducting a network meta‐analysis (Ioannidis, 2009) to synthesise the reviews that performed meta‐analysis but our sample of meta‐analyses will be too small to allow a meaningful network meta‐analysis.

15
http://methods.cochrane.org/sites/methods.cochrane.org.equity/files/public/uploads/EquityChecklist2012.pdf

## Appendix 1 ‐ Search strategies

1. **Academic Search Complete (Ebsco) – Searched 10^th^ November 2017**

S23 S11 AND S16 AND S21 Limiters ‐ Published Date: 20100101‐20181231

**366 hits**

S22 S16 AND S21

2,637

S21 S17 OR S18 OR S19 OR S20

216,396

S20 TI ((mhealth or “mobile health” or m‐health)) OR AB ((mhealth or “mobile health” or m‐health)) OR SU ((mhealth or “mobile health” or m‐health))

1,504

S19 SU (micro‐finance OR “micro finance” OR microfinance OR micro‐loan* OR microloan* OR “micro loan*” OR microleas* OR micro‐leas* OR “micro leas*” OR microlending OR micro‐lending OR “micro lending” OR microinsurance OR micro‐insurance OR “micro insurance” OR “microgroup lending” OR microfranchis* OR micro‐franchis* OR “micro franchis*” OR “micro credit*” OR microcredit* OR micro‐credit* OR “micro enterprise*” OR microenterprise* OR micro‐enterprise* OR “micro entrepreneur*” OR microentrepreneur* OR micro‐entrepreneur* OR saving* OR micro‐saving* OR microsaving* OR “Smallholder financ*” OR “rural financ*” OR “rural credit” OR ROSCAs OR SHGs OR “group lending” OR “community savings” or “small loan*” or “small lend*” or ((bank or credit*) N3 cooperat*) or ((credit or loan* or lend*) N3 program*) or (community N3 (bank* or saving* or loan* or lend*)) or “income generat*” or grameen OR ROSCA* OR stokvel* OR ((financial OR economic) N2 (literacy OR education OR skills OR training OR knowledge OR capab*)) OR banking OR budgeting OR “money manag*” OR “consumption smoothing” OR rationing OR earmarking OR “bank account*” OR “youth account*” OR “lock box*” OR “piggy bank*” OR “saving box*” OR ((access* OR participat*) N3 (financ* OR credit OR saving* OR loan* OR lending)) OR “financial inclusion” OR “inclusive finance” OR fintech OR “mobile monies” OR M‐PESA OR “mobile banking” OR cashless)

66,273

S18 AB (micro‐finance OR “micro finance” OR microfinance OR micro‐loan* OR microloan* OR “micro loan*” OR microleas* OR micro‐leas* OR “micro leas*” OR microlending OR micro‐lending OR “micro lending” OR microinsurance OR micro‐insurance OR “micro insurance” OR “microgroup lending” OR microfranchis* OR micro‐franchis* OR “micro franchis*” OR “micro credit*” OR microcredit* OR micro‐credit* OR “micro enterprise*” OR microenterprise* OR micro‐enterprise* OR “micro entrepreneur*” OR microentrepreneur* OR micro‐entrepreneur* OR saving* OR micro‐saving* OR microsaving* OR “Smallholder financ*” OR “rural financ*” OR “rural credit” OR SHGs OR “group lending” OR “community savings” or “small loan*” or “small lend*” or ((bank or credit*) N3 cooperat*) or ((credit or loan* or lend*) N3 program*) or (community N3 (bank* or saving* or loan* or lend*)) or “income generat*” or grameen OR ROSCA* OR stokvel* OR ((financial OR economic) N2 (literacy OR education OR skills OR training OR knowledge OR capab*)) OR banking OR budgeting OR “money manag*” OR “consumption smoothing” OR rationing OR earmarking OR “bank account*” OR “youth account*” OR “lock box*” OR “piggy bank*” OR “saving box*” OR ((access* OR participat*) N3 (financ* OR credit OR saving* OR loan* OR lending)) OR “financial inclusion” OR “inclusive finance” OR fintech OR “mobile monies” OR M‐PESA OR “mobile banking” OR cashless)

158,815

S17 TI (micro‐finance OR “micro finance” OR microfinance OR micro‐loan* OR microloan* OR “micro loan*” OR microleas* OR micro‐leas* OR “micro leas*” OR microlending OR micro‐lending OR “micro lending” OR microinsurance OR micro‐insurance OR “micro insurance” OR “microgroup lending” OR microfranchis* OR micro‐franchis* OR “micro franchis*” OR “micro credit*” OR microcredit* OR micro‐credit* OR “micro enterprise*” OR microenterprise* OR micro‐enterprise* OR “micro entrepreneur*” OR microentrepreneur* OR micro‐entrepreneur* OR saving* OR micro‐saving* OR microsaving* OR “Smallholder financ*” OR “rural financ*” OR “rural credit” OR SHGs OR “group lending” OR “community savings” or “small loan*” or “small lend*” or ((bank or credit*) N3 cooperat*) or ((credit or loan* or lend*) N3 program*) or (community N3 (bank* or saving* or loan* or lend*)) or “income generat*” or grameen OR ROSCA* OR stokvel* OR ((financial OR economic) N2 (literacy OR education OR skills OR training OR knowledge OR capab*)) OR banking OR budgeting OR “money manag*” OR “consumption smoothing” OR rationing OR earmarking OR “bank account*” OR “youth account*” OR “lock box*” OR “piggy bank*” OR “saving box*” OR ((access* OR participat*) N3 (financ* OR credit OR saving* OR loan* OR lending)) OR “financial inclusion” OR “inclusive finance” OR fintech OR “mobile monies” OR M‐PESA OR “mobile banking” OR cashless)

34,309

S16 S12 or S13 or S14 or S15

433,532

S15 TI ((“literature search” OR “database search” OR “bibliographic* search” OR “comprehensive search” OR “extensive search” OR “exhaustive search” OR “purposive search” OR “representative search” or “systemat* search”)) OR AB ((“literature search” OR “database search” OR “bibliographic* search” OR “comprehensive search” OR “extensive search” OR “exhaustive search” OR “purposive search” OR “representative search” or “systemat* search”)) OR SU ((“literature search” OR “database search” OR “bibliographic* search” OR “comprehensive search” OR “extensive search” OR “exhaustive search” OR “purposive search” OR “representative search” or “systemat* search”))

25,968

S14 TI ((review N3 (effectiveness OR effects OR systemat* OR synth* OR integrat* OR map* OR methodologic* OR quantitative OR evidence OR literature))) OR AB ((review N3 (effectiveness OR effects OR systemat* OR synth* OR integrat* OR map* OR methodologic* OR quantitative OR evidence OR literature))) OR SU ((review N3 (effectiveness OR effects OR systemat* OR synth* OR integrat* OR map* OR methodologic* OR quantitative OR evidence OR literature)))

223,448

S13 TI ((“Meta regression” OR “meta synth*” OR “meta‐synth*” OR “meta analy*” OR “metaanaly*” OR “meta‐analy*” OR “metanaly*” OR “Metaregression” OR “Meta‐regression” OR “Methodologic* overview” OR “pool* analys*” OR “pool* data” OR “Quantitative* overview” OR “research integration”)) OR AB ((“Meta regression” OR “meta synth*” OR “meta‐synth*” OR “meta analy*” OR “metaanaly*” OR “meta‐analy*” OR “metanaly*” OR “Metaregression” OR “Meta‐regression” OR “Methodologic* overview” OR “pool* analys*” OR “pool* data” OR “Quantitative* overview” OR “research integration”)) OR SU ((“Meta regression” OR “meta synth*” OR “meta‐synth*” OR “meta analy*” OR “metaanaly*” OR “meta‐analy*” OR “metanaly*” OR “Metaregression” OR “Meta‐regression” OR “Methodologic* overview” OR “pool* analys*” OR “pool* data” OR “Quantitative* overview” OR “research integration”))

81,114

S12 TI (((Systematic* OR synthes*) N3 (Research OR evaluation* OR finding* OR thematic* OR report OR descriptive OR explanatory OR narrative OR meta* OR review* OR data OR literature OR studies OR evidence OR map OR quantitative OR study OR studies OR paper OR impact OR impacts OR effect* OR compar*))) OR AB (((Systematic* OR synthes*) N3 (Research OR evaluation* OR finding* OR thematic* OR report OR descriptive OR explanatory OR narrative OR meta* OR review* OR data OR literature OR studies OR evidence OR map OR quantitative OR study OR studies OR paper OR impact OR impacts OR effect* OR compar*))) OR SU (((Systematic* OR synthes*) N3 (Research OR evaluation* OR finding* OR thematic* OR report OR descriptive OR explanatory OR narrative OR meta* OR review* OR data OR literature OR studies OR evidence OR map OR quantitative OR study OR studies OR paper OR impact OR impacts OR effect* OR compar*)))

238,573

S11 S1 OR S2 OR S3 OR S4 OR S5 OR S6 OR S7 OR S8 OR S9 OR S10

2,444,336

S10 TI (Africa or Asia or Caribbean or “West Indies” or “South America” or “Latin America” or “Central America” OR “Middle East”) OR AB (Africa or Asia or Caribbean or “West Indies” or “South America” or “Latin America” or “Central America” OR “Middle East”) OR SU (Africa or Asia or Caribbean or “West Indies” or “South America” or “Latin America” or “Central America” OR “Middle East”) OR GE (Africa or Asia or Caribbean or “West Indies” or “South America” or “Latin America” or “Central America” OR “Middle East”)

453,330

S9 TI ((“transitional country” or “transitional countries”)) OR AB ((“transitional country” or “transitional countries”)) OR SU ((“transitional country” or “transitional countries”))

227

S8 TI ((lmic or lmics or “third world” or “lami country” or “lami countries”)) OR AB ((lmic or lmics or “third world” or “lami country” or “lami countries”)) OR SU ((lmic or lmics or “third world” or “lami country” or “lami countries”))

10,222

S7 TI (low N3 middle N3 countr*) OR AB (low N3 middle N3 countr*) OR SU (low N3 middle N3 countr*)

6,211

S6 TI (low* N1 (gdp or gnp or “gross domestic” or “gross national”)) OR AB (low* N1 (gdp or gnp or “gross domestic” or “gross national”)) OR SU (low* N1 (gdp or gnp or “gross domestic” or “gross national”))

256

S5 TI ((developing or less* N1 developed or “under developed” or underdeveloped or “middle income” or low* N1 income) N1 (economy or economies)) OR AB ((developing or less* N1 developed or “under developed” or underdeveloped or “middle income” or low* N1 income) N1 (economy or economies)) OR SU ((developing or less* N1 developed or “under developed” or underdeveloped or “middle income” or low* N1 income) N1 (economy or economies))

1,996

S4 TI ((developing or less* N1 developed or “under developed” or underdeveloped or “middle income” or low* N1 income or underserved or “under served” or deprived or poor*) N1 (countr* or nation* or population* or world)) OR AB ((developing or less* N1 developed or “under developed” or underdeveloped or “middle income” or low* N1 income or underserved or “under served” or deprived or poor*) N1 (countr* or nation* or population* or world)) OR SU ((developing or less* N1 developed or “under developed” or underdeveloped or “middle income” or low* N1 income or underserved or “under served” or deprived or poor*) N1 (countr* or nation* or population* or world))

94,574

S3 AB Afghanistan OR Albania OR Algeria OR Angola OR Antigua OR Barbuda OR Argentina OR Armenia OR Armenian OR Aruba OR Azerbaijan OR Bahrain OR Bangladesh OR Barbados OR Benin OR Belize OR Bhutan OR Bolivia OR Botswana OR Brazil OR Brasil OR “Burkina Faso” OR “Burkina Fasso” OR “Upper Volta” OR Burundi OR Urundi OR Cambodia OR “Khmer Republic” OR Kampuchea OR Cameroon OR Cameroons OR Cameron OR Camerons OR “Cape Verde” OR “Central African Republic” OR Chad OR Chile OR China OR Colombia OR Comoros OR “Comoro Islands” OR Comores OR Mayotte OR Congo OR Zaire OR “Costa Rica” OR “Cote d'Ivoire” OR “Ivory Coast” OR Cuba OR “Djibouti” OR “French Somaliland” OR Dominica OR “Dominican Republic” OR “East Timor” OR “East Timur” OR “Timor Leste” OR Ecuador OR Egypt OR “United Arab Republic” OR “El Salvador” OR Eritrea OR Ethiopia OR Fiji OR Gabon OR “Gabonese Republic” OR Gambia OR Gaza OR “Georgia Republic” OR “Georgian Republic” OR Ghana OR “Gold Coast” OR Grenada OR Guatemala OR Guinea OR Guam OR Guiana OR Guyana OR Haiti OR Honduras OR India OR Maldives OR Indonesia OR Iran OR Iraq OR Jamaica OR Jordan OR Kazakhstan OR Kazakh OR Kenya OR Kiribati OR Korea OR Kosovo OR Kyrgyzstan OR Kirghizia OR “Kyrgyz Republic” OR Kirghiz OR Kirgizstan OR “Lao PDR” OR Laos OR Lebanon OR Lesotho OR Basutoland OR Liberia OR Libya OR Madagascar OR “Malagasy Republic” OR Malaysia OR Malaya OR Malay OR Sabah OR Sarawak OR Malawi OR Nyasaland OR Mali OR “Marshall Islands” OR Mauritania OR Mauritius OR “Agalega Islands” OR Mexico OR Micronesia OR “Middle East” OR Moldova OR Moldovia OR Moldovian OR Mongolia OR Montenegro OR Morocco OR Ifni OR Mozambique OR Myanmar OR Myanma OR Burma OR Namibia OR Nepal OR Antilles OR “New Caledonia” OR Nicaragua OR Niger OR Nigeria OR “Mariana Islands” OR Oman OR Muscat OR Pakistan OR Palau OR Palestine OR Panama OR Paraguay OR Peru OR Philippines OR Philipines OR Phillipines OR Phillippines OR “Puerto Rico” OR Rwanda OR Ruanda OR “Saint Kitts” OR “St Kitts” OR Nevis OR “Saint Lucia” OR “St Lucia” OR “Saint Vincent” OR “St Vincent” OR “Grenadines” OR “Samoa” OR “Samoan Islands” OR “Navigator Island” OR “Navigator Islands” OR “Sao Tome” OR “Saudi Arabia” OR Senegal OR Seychelles OR “Sierra Leone” OR “Sri Lanka” OR “Solomon Islands” OR Somalia OR Sudan OR Suriname OR Surinam OR Swaziland OR Syria OR Tajikistan OR Tadzhikistan OR Tadjikistan OR Tadzhik OR Tanzania OR Thailand OR Togo OR “Togolese Republic” OR Tonga OR Trinidad OR Tobago OR Tunisia OR Turkey OR Turkmenistan OR Turkmen OR Uganda OR Ukraine OR Uruguay OR Uzbekistan OR Uzbek OR Vanuatu OR “New Hebrides” OR Venezuela OR Vietnam OR “Viet Nam” OR “West Bank” OR Yemen OR Zambia OR Zimbabwe OR Jamahiriya OR Jamahiryria OR Libia OR Mocambique OR Principe OR Syrian OR “Indian Ocean” OR Melanesia OR “Western Sahara”

1,733,865

S2 TI Afghanistan OR Albania OR Algeria OR Angola OR Antigua OR Barbuda OR Argentina OR Armenia OR Armenian OR Aruba OR Azerbaijan OR Bahrain OR Bangladesh OR Barbados OR Benin OR Belize OR Bhutan OR Bolivia OR Botswana OR Brazil OR Brasil OR “Burkina Faso” OR “Burkina Fasso” OR “Upper Volta” OR Burundi OR Urundi OR Cambodia OR “Khmer Republic” OR Kampuchea OR Cameroon OR Cameroons OR Cameron OR Camerons OR “Cape Verde” OR “Central African Republic” OR Chad OR Chile OR China OR Colombia OR Comoros OR “Comoro Islands” OR Comores OR Mayotte OR Congo OR Zaire OR “Costa Rica” OR “Cote d'Ivoire” OR “Ivory Coast” OR Cuba OR “Djibouti” OR “French Somaliland” OR Dominica OR “Dominican Republic” OR “East Timor” OR “East Timur” OR “Timor Leste” OR Ecuador OR Egypt OR “United Arab Republic” OR “El Salvador” OR Eritrea OR Ethiopia OR Fiji OR Gabon OR “Gabonese Republic” OR Gambia OR Gaza OR “Georgia Republic” OR “Georgian Republic” OR Ghana OR “Gold Coast” OR Grenada OR Guatemala OR Guinea OR Guam OR Guiana OR Guyana OR Haiti OR Honduras OR India OR Maldives OR Indonesia OR Iran OR Iraq OR Jamaica OR Jordan OR Kazakhstan OR Kazakh OR Kenya OR Kiribati OR Korea OR Kosovo OR Kyrgyzstan OR Kirghizia OR “Kyrgyz Republic” OR Kirghiz OR Kirgizstan OR “Lao PDR” OR Laos OR Lebanon OR Lesotho OR Basutoland OR Liberia OR Libya OR Madagascar OR “Malagasy Republic” OR Malaysia OR Malaya OR Malay OR Sabah OR Sarawak OR Malawi OR Nyasaland OR Mali OR “Marshall Islands” OR Mauritania OR Mauritius OR “Agalega Islands” OR Mexico OR Micronesia OR “Middle East” OR Moldova OR Moldovia OR Moldovian OR Mongolia OR Montenegro OR Morocco OR Ifni OR Mozambique OR Myanmar OR Myanma OR Burma OR Namibia OR Nepal OR Antilles OR “New Caledonia” OR Nicaragua OR Niger OR Nigeria OR “Mariana Islands” OR Oman OR Muscat OR Pakistan OR Palau OR Palestine OR Panama OR Paraguay OR Peru OR Philippines OR Philipines OR Phillipines OR Phillippines OR “Puerto Rico” OR Rwanda OR Ruanda OR “Saint Kitts” OR “St Kitts” OR Nevis OR “Saint Lucia” OR “St Lucia” OR “Saint Vincent” OR “St Vincent” OR “Grenadines” OR “Samoa” OR “Samoan Islands” OR “Navigator Island” OR “Navigator Islands” OR “Sao Tome” OR “Saudi Arabia” OR Senegal OR Seychelles OR “Sierra Leone” OR “Sri Lanka” OR “Solomon Islands” OR Somalia OR Sudan OR Suriname OR Surinam OR Swaziland OR Syria OR Tajikistan OR Tadzhikistan OR Tadjikistan OR Tadzhik OR Tanzania OR Thailand OR Togo OR “Togolese Republic” OR Tonga OR Trinidad OR Tobago OR Tunisia OR Turkey OR Turkmenistan OR Turkmen OR Uganda OR Ukraine OR Uruguay OR Uzbekistan OR Uzbek OR Vanuatu OR “New Hebrides” OR Venezuela OR Vietnam OR “Viet Nam” OR “West Bank” OR Yemen OR Zambia OR Zimbabwe OR Jamahiriya OR Jamahiryria OR Libia OR Mocambique OR Principe OR Syrian OR “Indian Ocean” OR Melanesia OR “Western Sahara”

837,520

S1 SU Afghanistan OR Albania OR Algeria OR Angola OR Antigua OR Barbuda OR Argentina OR Armenia OR Armenian OR Aruba OR Azerbaijan OR Bahrain OR Bangladesh OR Barbados OR Benin OR Belize OR Bhutan OR Bolivia OR Botswana OR Brazil OR Brasil OR “Burkina Faso” OR “Burkina Fasso” OR “Upper Volta” OR Burundi OR Urundi OR Cambodia OR “Khmer Republic” OR Kampuchea OR Cameroon OR Cameroons OR Cameron OR Camerons OR “Cape Verde” OR “Central African Republic” OR Chad OR Chile OR China OR Colombia OR Comoros OR “Comoro Islands” OR Comores OR Mayotte OR Congo OR Zaire OR “Costa Rica” OR “Cote d'Ivoire” OR “Ivory Coast” OR Cuba OR “Djibouti” OR “French Somaliland” OR Dominica OR “Dominican Republic” OR “East Timor” OR “East Timur” OR “Timor Leste” OR Ecuador OR Egypt OR “United Arab Republic” OR “El Salvador” OR Eritrea OR Ethiopia OR Fiji OR Gabon OR “Gabonese Republic” OR Gambia OR Gaza OR “Georgia Republic” OR “Georgian Republic” OR Ghana OR “Gold Coast” OR Grenada OR Guatemala OR Guinea OR Guam OR Guiana OR Guyana OR Haiti OR Honduras OR India OR Maldives OR Indonesia OR Iran OR Iraq OR Jamaica OR Jordan OR Kazakhstan OR Kazakh OR Kenya OR Kiribati OR Korea OR Kosovo OR Kyrgyzstan OR Kirghizia OR “Kyrgyz Republic” OR Kirghiz OR Kirgizstan OR “Lao PDR” OR Laos OR Lebanon OR Lesotho OR Basutoland OR Liberia OR Libya OR Madagascar OR “Malagasy Republic” OR Malaysia OR Malaya OR Malay OR Sabah OR Sarawak OR Malawi OR Nyasaland OR Mali OR “Marshall Islands” OR Mauritania OR Mauritius OR “Agalega Islands” OR Mexico OR Micronesia OR “Middle East” OR Moldova OR Moldovia OR Moldovian OR Mongolia OR Montenegro OR Morocco OR Ifni OR Mozambique OR Myanmar OR Myanma OR Burma OR Namibia OR Nepal OR Antilles OR “New Caledonia” OR Nicaragua OR Niger OR Nigeria OR “Mariana Islands” OR Oman OR Muscat OR Pakistan OR Palau OR Palestine OR Panama OR Paraguay OR Peru OR Philippines OR Philipines OR Phillipines OR Phillippines OR “Puerto Rico” OR Rwanda OR Ruanda OR “Saint Kitts” OR “St Kitts” OR Nevis OR “Saint Lucia” OR “St Lucia” OR “Saint Vincent” OR “St Vincent” OR “Grenadines” OR “Samoa” OR “Samoan Islands” OR “Navigator Island” OR “Navigator Islands” OR “Sao Tome” OR “Saudi Arabia” OR Senegal OR Seychelles OR “Sierra Leone” OR “Sri Lanka” OR “Solomon Islands” OR Somalia OR Sudan OR Suriname OR Surinam OR Swaziland OR Syria OR Tajikistan OR Tadzhikistan OR Tadjikistan OR Tadzhik OR Tanzania OR Thailand OR Togo OR “Togolese Republic” OR Tonga OR Trinidad OR Tobago OR Tunisia OR Turkey OR Turkmenistan OR Turkmen OR Uganda OR Ukraine OR Uruguay OR Uzbekistan OR Uzbek OR Vanuatu OR “New Hebrides” OR Venezuela OR Vietnam OR “Viet Nam” OR “West Bank” OR Yemen OR Zambia OR Zimbabwe OR Jamahiriya OR Jamahiryria OR Libia OR Mocambique OR Principe OR Syrian OR “Indian Ocean” OR Melanesia OR “Western Sahara”

1,272,552

**Ebsco Discovery Service ‐ Searched 10^th^ November 2017**

Strategy for Academic Search Complete (above) used – limited to:

2. **Econlit (510 hits)**
3. **Repec (238 hits)**
4. **World Bank e‐Library (40 hits)**
5. **Scopus – Searched 10^th^ November 2017**

(((TITLE‐ABS‐KEY (mhealth OR “mobile health” OR “m health” OR m‐health))) OR ((TITLE‐ABS‐KEY (micro‐finance OR “micro finance” OR microfinance OR micro‐loan* OR microloan* OR “micro loan*” OR microleas* OR micro‐leas* OR “micro leas*” OR microlending OR micro‐lending OR “micro lending” OR microinsurance OR micro‐insurance OR “micro insurance” OR “microgroup lending” OR microfranchis* OR micro‐franchis* OR “micro franchis*” OR “micro credit*” OR microcredit* OR micro‐credit* OR “micro enterprise*” OR microenterprise* OR micro‐enterprise* OR “micro entrepreneur*” OR microentrepreneur* OR micro‐entrepreneur* OR saving* OR micro‐saving* OR microsaving* OR “Smallholder financ*” OR “rural financ*” OR “rural credit” OR roscas OR shgs OR “group lending” OR “community savings” OR “small loan*” OR “small lend*” OR ((bank* OR credit*) W/3 cooperat*) OR ((credit OR loan* OR lend*) W/3 program*) OR (community W/3 (bank* OR saving* OR loan* OR lend*)) OR “income generat*” OR grameen OR rosca* OR stokvel* OR ((financial OR economic) W/2 (literacy OR education OR skills OR training OR knowledge OR capab*)) OR banking OR budgeting OR “money manag*” OR “consumption smoothing” OR rationing OR earmarking OR “bank account*” OR “youth account*” OR “lock box*” OR “piggy bank*” OR “saving box*” OR ((access* OR participat*) W/3 (financ* OR credit OR saving* OR loan* OR lending)) OR “financial inclusion” OR “inclusive finance” OR fintech OR “mobile monies” OR m‐pesa OR “mobile banking” OR cashless)))) AND (((TITLE‐ABS‐KEY (“literature search” OR “database search” OR “bibliographic* search” OR “comprehensive search” OR “extensive search” OR “exhaustive search” OR “purposive search” OR “representative search” OR “systemat* search”))) OR ((TITLE‐ABS‐KEY (review W/3 (effectiveness OR effects OR systemat* OR synth* OR integrat* OR map* OR methodologic* OR quantitative OR evidence OR literature)))) OR ((TITLE‐ABS‐KEY (“Meta regression” OR “meta synth*” OR “meta‐synth*” OR “meta analy*” OR “metaanaly*” OR “meta‐analy*” OR “metanaly*” OR “Metaregression” OR “Meta‐regression” OR “Methodologic* overview” OR “pool* analys*” OR “pool* data” OR “Quantitative* overview” OR “research integration”))) OR ((TITLE‐ABS‐KEY ((systematic* OR synthes*) W/3 (research OR evaluation* OR finding* OR thematic* OR report OR descriptive OR explanatory OR narrative OR meta* OR review* OR data OR literature OR studies OR evidence OR map OR quantitative OR study OR studies OR paper OR impact OR impacts OR effect* OR compar*))))) AND (((TITLE‐ABS‐KEY (low* W/1 (gdp OR gnp OR “gross domestic” OR “gross national”)))) OR ((TITLE‐ABS‐KEY ((developing OR less* W/1 developed OR “under developed” OR underdeveloped OR “middle income” OR low* W/1 income) W/1 (economy OR economies)))) OR ((TITLE‐ABS‐KEY ((developing OR (less* W/1 developed) OR “under developed” OR underdeveloped OR “middle income” OR (low* W/1 income) OR underserved OR “under served” OR deprived OR poor*) W/1 (countr* OR nation* OR population* OR world)))) OR ((TITLE‐ABS‐KEY (afghanistan OR albania OR algeria OR angola OR antigua OR barbuda OR argentina OR armenia OR armenian OR aruba OR azerbaijan OR bahrain OR bangladesh OR barbados OR benin OR belize OR bhutan OR bolivia OR botswana OR brazil OR brasil OR “Burkina Faso” OR “Burkina Fasso” OR “Upper Volta” OR burundi OR urundi OR cambodia OR “Khmer Republic” OR kampuchea OR cameroon OR cameroons OR cameron OR camerons OR “Cape Verde” OR “Central African Republic” OR chad OR chile OR china OR colombia OR comoros OR “Comoro Islands” OR comores OR mayotte OR congo OR zaire OR “Costa Rica” OR “Cote d'Ivoire” OR “Ivory Coast” OR cuba OR “Djibouti” OR “French Somaliland” OR dominica OR “Dominican Republic” OR “East Timor” OR “East Timur” OR “Timor Leste” OR ecuador OR egypt OR “United Arab Republic” OR “El Salvador” OR eritrea OR ethiopia OR fiji OR gabon OR “Gabonese Republic” OR gambia OR gaza OR “Georgia Republic” OR “Georgian Republic” OR ghana OR “Gold Coast” OR grenada OR guatemala OR guinea OR guam OR guiana OR guyana OR haiti OR honduras OR india OR maldives OR indonesia OR iran OR iraq OR jamaica OR jordan OR kazakhstan OR kazakh OR kenya OR kiribati OR korea OR kosovo OR kyrgyzstan OR kirghizia OR “Kyrgyz Republic” OR kirghiz OR kirgizstan OR “Lao PDR” OR laos OR lebanon OR lesotho OR basutoland OR liberia OR libya OR madagascar OR “Malagasy Republic” OR malaysia OR malaya OR malay OR sabah OR sarawak OR malawi OR nyasaland OR mali OR “Marshall Islands” OR mauritania OR mauritius OR “Agalega Islands” OR mexico OR micronesia OR “Middle East” OR moldova OR moldovia OR moldovian OR mongolia OR montenegro OR morocco OR ifni OR mozambique OR myanmar OR myanma OR burma OR namibia OR nepal OR antilles OR “New Caledonia” OR nicaragua OR niger OR nigeria OR “Mariana Islands” OR oman OR muscat OR pakistan OR palau OR palestine OR panama OR paraguay OR peru OR philippines OR philipines OR phillipines OR phillippines OR “Puerto Rico” OR rwanda OR ruanda OR “Saint Kitts” OR “St Kitts” OR nevis OR “Saint Lucia” OR “St Lucia” OR “Saint Vincent” OR “St Vincent” OR “Grenadines” OR “Samoa” OR “Samoan Islands” OR “Navigator Island” OR “Navigator Islands” OR “Sao Tome” OR “Saudi Arabia” OR senegal OR seychelles OR “Sierra Leone” OR “Sri Lanka” OR “Solomon Islands” OR somalia OR sudan OR suriname OR surinam OR swaziland OR syria OR tajikistan OR tadzhikistan OR tadjikistan OR tadzhik OR tanzania OR thailand OR togo OR “Togolese Republic” OR tonga OR trinidad OR tobago OR tunisia OR turkey OR turkmenistan OR turkmen OR uganda OR ukraine OR uruguay OR uzbekistan OR uzbek OR vanuatu OR “New Hebrides” OR venezuela OR vietnam OR “Viet Nam” OR “West Bank” OR yemen OR zambia OR zimbabwe OR jamahiriya OR jamahiryria OR libia OR mocambique OR principe OR syrian OR “Indian Ocean” OR melanesia OR “Western Sahara”))) OR (((TITLE‐ABS‐KEY (africa OR asia OR caribbean OR “West Indies” OR “South America” OR “Latin America” OR “Central America” OR “Middle East”))) OR ((TITLE‐ABS‐KEY (“transitional country” OR “transitional countries”))) OR ((TITLE‐ABS‐KEY (lmic OR lmics OR “third world” OR “lami country” OR “lami countries”))) OR ((TITLE‐ABS‐KEY (low W/3 middle W/3 countr*))))) AND (LIMIT‐TO (PUBYEAR, 2018) OR LIMIT‐TO (PUBYEAR, 2017) OR LIMIT‐TO (PUBYEAR, 2016) OR LIMIT‐TO (PUBYEAR, 2015) OR LIMIT‐TO (PUBYEAR, 2014) OR LIMIT‐TO (PUBYEAR, 2013) OR LIMIT‐TO (PUBYEAR, 2012) OR LIMIT‐TO (PUBYEAR, 2011) OR LIMIT‐TO (PUBYEAR, 2010)) 19 (((TITLE‐ABS‐KEY (mhealth OR “mobile health” OR “m health” OR m‐health))) OR ((TITLE‐ABS‐KEY (micro‐finance OR “micro finance” OR microfinance OR micro‐loan* OR microloan* OR “micro loan*” OR microleas* OR micro‐leas* OR “micro leas*” OR microlending OR micro‐lending OR “micro lending” OR microinsurance OR micro‐insurance OR “micro insurance” OR “microgroup lending” OR microfranchis* OR micro‐franchis* OR “micro franchis*” OR “micro credit*” OR microcredit* OR micro‐credit* OR “micro enterprise*” OR microenterprise* OR micro‐enterprise* OR “micro entrepreneur*” OR microentrepreneur* OR micro‐entrepreneur* OR saving* OR micro‐saving* OR microsaving* OR “Smallholder financ*” OR “rural financ*” OR “rural credit” OR roscas OR shgs OR “group lending” OR “community savings” OR “small loan*” OR “small lend*” OR ((bank* OR credit*) W/3 cooperat*) OR ((credit OR loan* OR lend*) W/3 program*) OR (community W/3 (bank* OR saving* OR loan* OR lend*)) OR “income generat*” OR grameen OR rosca* OR stokvel* OR ((financial OR economic) W/2 (literacy OR education OR skills OR training OR knowledge OR capab*)) OR banking OR budgeting OR “money manag*” OR “consumption smoothing” OR rationing OR earmarking OR “bank account*” OR “youth account*” OR “lock box*” OR “piggy bank*” OR “saving box*” OR ((access* OR participat*) W/3 (financ* OR credit OR saving* OR loan* OR lending)) OR “financial inclusion” OR “inclusive finance” OR fintech OR “mobile monies” OR m‐pesa OR “mobile banking” OR cashless)))) AND (((TITLE‐ABS‐KEY (“literature search” OR “database search” OR “bibliographic* search” OR comprehensive AND search “ OR extensive search” OR “exhaustive search” OR “purposive search” OR “representative search” OR “systemat* search”))) OR ((TITLE‐ABS‐KEY (review W/3 (effectiveness OR effects OR systemat* OR synth* OR integrat* OR map* OR methodologic* OR quantitative OR evidence OR literature)))) OR ((TITLE‐ABS‐KEY (“Meta regression” OR “meta synth*” OR “meta‐synth*” OR “meta analy*” OR “metaanaly*” OR “meta‐analy*” OR “metanaly*” OR “Metaregression” OR “Meta‐regression” OR “Methodologic* overview” OR “pool* analys*” OR “pool* data” OR “Quantitative* overview” OR “research integration”))) OR ((TITLE‐ABS‐KEY ((systematic* OR synthes*) W/3 (research OR evaluation* OR finding* OR thematic* OR report OR descriptive OR explanatory OR narrative OR meta* OR review* OR data OR literature OR studies OR evidence OR map OR quantitative OR study OR studies OR paper OR impact OR impacts OR effect* OR compar*))))) AND (((TITLE‐ABS‐KEY (low* W/1 (gdp OR gnp OR “gross domestic” OR “gross national”)))) OR ((TITLE‐ABS‐KEY ((developing OR less* W/1 developed OR “under developed” OR underdeveloped OR “middle income” OR low* W/1 income) W/1 (economy OR economies)))) OR ((TITLE‐ABS‐KEY ((developing OR (less* W/1 developed) OR “under developed” OR underdeveloped OR “middle income” OR (low* W/1 income) OR underserved OR “under served” OR deprived OR poor*) W/1 (countr* OR nation* OR population* OR world)))) OR ((TITLE‐ABS‐KEY (afghanistan OR albania OR algeria OR angola OR antigua OR barbuda OR argentina OR armenia OR armenian OR aruba OR azerbaijan OR bahrain OR bangladesh OR barbados OR benin OR belize OR bhutan OR bolivia OR botswana OR brazil OR brasil OR “Burkina Faso” OR “Burkina Fasso” OR “Upper Volta” OR burundi OR urundi OR cambodia OR “Khmer Republic” OR kampuchea OR cameroon OR cameroons OR cameron OR camerons OR “Cape Verde” OR “Central African Republic” OR chad OR chile OR china OR colombia OR comoros OR “Comoro Islands” OR comores OR mayotte OR congo OR zaire OR “Costa Rica” OR “Cote d'Ivoire” OR “Ivory Coast” OR cuba OR “Djibouti” OR “French Somaliland” OR dominica OR “Dominican Republic” OR “East Timor” OR “East Timur” OR “Timor Leste” OR ecuador OR egypt OR “United Arab Republic” OR “El Salvador” OR eritrea OR ethiopia OR fiji OR gabon OR “Gabonese Republic” OR gambia OR gaza OR “Georgia Republic” OR “Georgian Republic” OR ghana OR “Gold Coast” OR grenada OR guatemala OR guinea OR guam OR guiana OR guyana OR haiti OR honduras OR india OR maldives OR indonesia OR iran OR iraq OR jamaica OR jordan OR kazakhstan OR kazakh OR kenya OR kiribati OR korea OR kosovo OR kyrgyzstan OR kirghizia OR “Kyrgyz Republic” OR kirghiz OR kirgizstan OR “Lao PDR” OR laos OR lebanon OR lesotho OR basutoland OR liberia OR libya OR madagascar OR “Malagasy Republic” OR malaysia OR malaya OR malay OR sabah OR sarawak OR malawi OR nyasaland OR mali OR “Marshall Islands” OR mauritania OR mauritius OR “Agalega Islands” OR mexico OR micronesia OR “Middle East” OR moldova OR moldovia OR moldovian OR mongolia OR montenegro OR morocco OR ifni OR mozambique OR myanmar OR myanma OR burma OR namibia OR nepal OR antilles OR “New Caledonia” OR nicaragua OR niger OR nigeria OR “Mariana Islands” OR oman OR muscat OR pakistan OR palau OR palestine OR panama OR paraguay OR peru OR philippines OR philipines OR phillipines OR phillippines OR “Puerto Rico” OR rwanda OR ruanda OR “Saint Kitts” OR “St Kitts” OR nevis OR “Saint Lucia” OR “St Lucia” OR “Saint Vincent” OR “St Vincent” OR “Grenadines” OR “Samoa” OR “Samoan Islands” OR “Navigator Island” OR “Navigator Islands” OR “Sao Tome” OR “Saudi Arabia” OR senegal OR seychelles OR “Sierra Leone” OR “Sri Lanka” OR “Solomon Islands” OR somalia OR sudan OR suriname OR surinam OR swaziland OR syria OR tajikistan OR tadzhikistan OR tadjikistan OR tadzhik OR tanzania OR thailand OR togo OR “Togolese Republic” OR tonga OR trinidad OR tobago OR tunisia OR turkey OR turkmenistan OR turkmen OR uganda OR ukraine OR uruguay OR uzbekistan OR uzbek OR vanuatu OR “New Hebrides” OR venezuela OR vietnam OR “Viet Nam” OR “West Bank” OR yemen OR zambia OR zimbabwe OR jamahiriya OR jamahiryria OR libia OR mocambique OR principe OR syrian OR “Indian Ocean” OR melanesia OR “Western Sahara”))) OR (((TITLE‐ABS‐KEY (africa OR asia OR caribbean OR “West Indies” OR “South America” OR “Latin America” OR “Central America” OR “Middle East”))) OR ((TITLE‐ABS‐KEY (“transitional country” OR “transitional countries”))) OR ((TITLE‐ABS‐KEY (lmic OR lmics OR “third world” OR “lami country” OR “lami countries”))) OR ((TITLE‐ABS‐KEY (low W/3 middle W/3 countr*))))) .

**1035 hits**

6. **Web of Science – Searched 14^th^ November 2017**

# 19 **2,014 hits**

#18 AND #8 AND #3 Indexes=SCI‐EXPANDED, SSCI, A&HCI Timespan=2010‐2017

# 18 5,049,601

#17 OR #16 OR #15 OR #14 OR #13 OR #12 OR #11 OR #10 OR #9

# 17 4,637,072

CU=(Afghanistan OR Albania OR Algeria OR Angola OR Antigua OR Barbuda OR Argentina OR Armenia OR Armenian OR Aruba OR Azerbaijan OR Bahrain OR Bangladesh OR Barbados OR Benin OR Belize OR Bhutan OR Bolivia OR Botswana OR Brazil OR Brasil OR “Burkina Faso” OR “Burkina Fasso” OR “Upper Volta” OR Burundi OR Urundi OR Cambodia OR “Khmer Republic” OR Kampuchea OR Cameroon OR Cameroons OR Cameron OR Camerons OR “Cape Verde” OR “Central African Republic” OR Chad OR Chile OR China OR Colombia OR Comoros OR “Comoro Islands” OR Comores OR Mayotte OR Congo OR Zaire OR “Costa Rica” OR “Cote d'Ivoire” OR “Ivory Coast” OR Cuba OR “Djibouti” OR “French Somaliland” OR Dominica OR “Dominican Republic” OR “East Timor” OR “East Timur” OR “Timor Leste” OR Ecuador OR Egypt OR “United Arab Republic” OR “El Salvador” OR Eritrea OR Ethiopia OR Fiji OR Gabon OR “Gabonese Republic” OR Gambia OR Gaza OR “Georgia Republic” OR “Georgian Republic” OR Ghana OR “Gold Coast” OR Grenada OR Guatemala OR Guinea OR Guam OR Guiana OR Guyana OR Haiti OR Honduras OR India OR Maldives OR Indonesia OR Iran OR Iraq OR Jamaica OR Jordan OR Kazakhstan OR Kazakh OR Kenya OR Kiribati OR Korea OR Kosovo OR Kyrgyzstan OR Kirghizia OR “Kyrgyz Republic” OR Kirghiz OR Kirgizstan OR “Lao PDR” OR Laos OR Lebanon OR Lesotho OR Basutoland OR Liberia OR Libya OR Madagascar OR “Malagasy Republic” OR Malaysia OR Malaya OR Malay OR Sabah OR Sarawak OR Malawi OR Nyasaland OR Mali OR “Marshall Islands” OR Mauritania OR Mauritius OR “Agalega Islands” OR Mexico OR Micronesia OR “Middle East” OR Moldova OR Moldovia OR Moldovian OR Mongolia OR Montenegro OR Morocco OR Ifni OR Mozambique OR Myanmar OR Myanma OR Burma OR Namibia OR Nepal OR Antilles OR “New Caledonia” OR Nicaragua OR Niger OR Nigeria OR “Mariana Islands” OR Oman OR Muscat OR Pakistan OR Palau OR Palestine OR Panama OR Paraguay OR Peru OR Philippines OR Philipines OR Phillipines OR Phillippines OR “Puerto Rico” OR Rwanda OR Ruanda OR “Saint Kitts” OR “St Kitts” OR Nevis OR “Saint Lucia” OR “St Lucia” OR “Saint Vincent” OR “St Vincent” OR “Grenadines” OR “Samoa” OR “Samoan Islands” OR “Navigator Island” OR “Navigator Islands” OR “Sao Tome” OR “Saudi Arabia” OR Senegal OR Seychelles OR “Sierra Leone” OR “Sri Lanka” OR “Solomon Islands” OR Somalia OR Sudan OR Suriname OR Surinam OR Swaziland OR Syria OR Tajikistan OR Tadzhikistan OR Tadjikistan OR Tadzhik OR Tanzania OR Thailand OR Togo OR “Togolese Republic” OR Tonga OR Trinidad OR Tobago OR Tunisia OR Turkey OR Turkmenistan OR Turkmen OR Uganda OR Ukraine OR Uruguay OR Uzbekistan OR Uzbek OR Vanuatu OR “New Hebrides” OR Venezuela OR Vietnam OR “Viet Nam” OR “West Bank” OR Yemen OR Zambia OR Zimbabwe OR Jamahiriya OR Jamahiryria OR Libia OR Mocambique OR Principe OR Syrian OR “Indian Ocean” OR Melanesia OR “Western Sahara”)

# 16 971,268

TS=(Afghanistan OR Albania OR Algeria OR Angola OR Antigua OR Barbuda OR Argentina OR Armenia OR Armenian OR Aruba OR Azerbaijan OR Bahrain OR Bangladesh OR Barbados OR Benin OR Belize OR Bhutan OR Bolivia OR Botswana OR Brazil OR Brasil OR “Burkina Faso” OR “Burkina Fasso” OR “Upper Volta” OR Burundi OR Urundi OR Cambodia OR “Khmer Republic” OR Kampuchea OR Cameroon OR Cameroons OR Cameron OR Camerons OR “Cape Verde” OR “Central African Republic” OR Chad OR Chile OR China OR Colombia OR Comoros OR “Comoro Islands” OR Comores OR Mayotte OR Congo OR Zaire OR “Costa Rica” OR “Cote d'Ivoire” OR “Ivory Coast” OR Cuba OR “Djibouti” OR “French Somaliland” OR Dominica OR “Dominican Republic” OR “East Timor” OR “East Timur” OR “Timor Leste” OR Ecuador OR Egypt OR “United Arab Republic” OR “El Salvador” OR Eritrea OR Ethiopia OR Fiji OR Gabon OR “Gabonese Republic” OR Gambia OR Gaza OR “Georgia Republic” OR “Georgian Republic” OR Ghana OR “Gold Coast” OR Grenada OR Guatemala OR Guinea OR Guam OR Guiana OR Guyana OR Haiti OR Honduras OR India OR Maldives OR Indonesia OR Iran OR Iraq OR Jamaica OR Jordan OR Kazakhstan OR Kazakh OR Kenya OR Kiribati OR Korea OR Kosovo OR Kyrgyzstan OR Kirghizia OR “Kyrgyz Republic” OR Kirghiz OR Kirgizstan OR “Lao PDR” OR Laos OR Lebanon OR Lesotho OR Basutoland OR Liberia OR Libya OR Madagascar OR “Malagasy Republic” OR Malaysia OR Malaya OR Malay OR Sabah OR Sarawak OR Malawi OR Nyasaland OR Mali OR “Marshall Islands” OR Mauritania OR Mauritius OR “Agalega Islands” OR Mexico OR Micronesia OR “Middle East” OR Moldova OR Moldovia OR Moldovian OR Mongolia OR Montenegro OR Morocco OR Ifni OR Mozambique OR Myanmar OR Myanma OR Burma OR Namibia OR Nepal OR Antilles OR “New Caledonia” OR Nicaragua OR Niger OR Nigeria OR “Mariana Islands” OR Oman OR Muscat OR Pakistan OR Palau OR Palestine OR Panama OR Paraguay OR Peru OR Philippines OR Philipines OR Phillipines OR Phillippines OR “Puerto Rico” OR Rwanda OR Ruanda OR “Saint Kitts” OR “St Kitts” OR Nevis OR “Saint Lucia” OR “St Lucia” OR “Saint Vincent” OR “St Vincent” OR “Grenadines” OR “Samoa” OR “Samoan Islands” OR “Navigator Island” OR “Navigator Islands” OR “Sao Tome” OR “Saudi Arabia” OR Senegal OR Seychelles OR “Sierra Leone” OR “Sri Lanka” OR “Solomon Islands” OR Somalia OR Sudan OR Suriname OR Surinam OR Swaziland OR Syria OR Tajikistan OR Tadzhikistan OR Tadjikistan OR Tadzhik OR Tanzania OR Thailand OR Togo OR “Togolese Republic” OR Tonga OR Trinidad OR Tobago OR Tunisia OR Turkey OR Turkmenistan OR Turkmen OR Uganda OR Ukraine OR Uruguay OR Uzbekistan OR Uzbek OR Vanuatu OR “New Hebrides” OR Venezuela OR Vietnam OR “Viet Nam” OR “West Bank” OR Yemen OR Zambia OR Zimbabwe OR Jamahiriya OR Jamahiryria OR Libia OR Mocambique OR Principe OR Syrian OR “Indian Ocean” OR Melanesia OR “Western Sahara”)

# 15 122,678

TS=((developing OR (less* NEAR developed) OR “under developed” OR underdeveloped OR “middle income” or (low* NEAR income)) NEAR (countr* or nation* or population* or world))

# 14 5,815

TS=((developing OR (less* NEAR developed) OR “under developed” OR underdeveloped OR “middle income” or (low* NEAR income)) NEAR (economy or economies))

# 13 1,137

TS=(low* NEAR (gdp OR gnp OR “gross domestic” OR “gross national”))

# 12 7,869

TS=(low NEAR/3 middle NEAR/3 countr*)

# 11 2,756

TS=(lmic OR lmics OR “third world” OR “lami country” OR “lami countries”)

# 10 169

TS=(“transitional country” OR “transitional countries”)

# 9 222,677

TS=(Africa OR Asia OR Caribbean OR “West Indies” OR “South America” OR “Latin America” OR “Central America” OR “Middle East”)

# 8 555,428

#7 OR #6 OR #5 OR #4

# 7 264,439

TS=(((Systematic* OR synthes*) NEAR/3 (Research OR evaluation* OR finding* OR thematic* OR report OR descriptive OR explanatory OR narrative OR meta* OR review* OR data OR literature OR evidence OR map OR quantitative OR study OR studies OR paper OR impact OR impacts OR effect* OR compar*)))

# 6 213,939

TS=(“Meta regression” OR “meta synth*” OR “meta‐synth*” OR “meta analy*” OR “metaanaly*” OR “meta‐analy*” OR “metanaly*” OR “Metaregression” OR “Meta‐regression” OR “Methodologic* overview” OR “pool* analys*” OR “pool* data” OR “Quantitative* overview” OR “research integration”)

# 5 223,106

TS=((review NEAR/3 (effectiveness OR effects OR systemat* OR synth* OR integrat* OR map* OR methodologic* OR quantitative OR evidence OR literature)))

# 4 32,845

TS=(“literature search” OR “database search” OR “bibliographic* search” OR “comprehensive search” OR “extensive search” OR “exhaustive search” OR “purposive search” OR “representative search” OR “systemat* search”)

# 3 160,645

#2 OR #1

# 2 157,779

TS=(micro‐finance OR “micro finance” OR microfinance OR micro‐loan* OR microloan* OR “micro loan*” OR microleas* OR micro‐leas* OR “micro leas*” OR microlending OR micro‐lending OR “micro lending” OR microinsurance OR micro‐insurance OR “micro insurance” OR “microgroup lending” OR microfranchis* OR micro‐franchis* OR “micro franchis*” OR “micro credit*” OR microcredit* OR micro‐credit* OR “micro enterprise*” OR microenterprise* OR micro‐enterprise* OR “micro entrepreneur*” OR microentrepreneur* OR micro‐entrepreneur* OR saving* OR micro‐saving* OR microsaving* OR “Smallholder financ*” OR “rural financ*” OR “rural credit” OR SHGs OR “group lending” OR “community savings” OR “small loan*” OR “small lend*” OR ((bank or credit*) NEAR/3 cooperat*) OR ((credit OR loan* OR lend*) NEAR/3 program*) OR (community NEAR/3 (bank* OR saving* OR loan* OR lend*)) OR “income generat*” OR grameen OR ROSCA* OR stokvel* OR ((financial OR economic) NEAR/2 (literacy OR education OR skills OR training OR knowledge OR capab*)) OR banking OR budgeting OR “money manag*” OR “consumption smoothing” OR rationing OR earmarking OR “bank account*” OR “youth account*” OR “lock box*” OR “piggy bank*” OR “saving box*” OR ((access* OR participat*) NEAR/3 (financ* OR credit OR saving* OR loan* OR lending)) OR “financial inclusion” OR “inclusive finance” OR fintech OR “mobile monies” OR M‐PESA OR “mobile banking” OR cashless)

# 1 2,919

TS=(mhealth OR “mobile health” OR m‐health)

7. **Business Source Premier (Ebsco) – Searched 18^th^ January 2018**

S23 S11 AND S16 AND S21 Limiters ‐ Published Date: 20100101‐20181231

**408 hits**

S22 S16 AND S21

1,142

S21 S17 OR S18 OR S19 OR S20

238,223

S20 TI ((mhealth or “mobile health” or m‐health)) OR AB ((mhealth or “mobile health” or m‐health)) OR SU ((mhealth or “mobile health” or m‐health))

629

S19 SU (micro‐finance OR “micro finance” OR microfinance OR micro‐loan* OR microloan* OR “micro loan*” OR microleas* OR micro‐leas* OR “micro leas*” OR microlending OR micro‐lending OR “micro lending” OR microinsurance OR micro‐insurance OR “micro insurance” OR “microgroup lending” OR microfranchis* OR micro‐franchis* OR “micro franchis*” OR “micro credit*” OR microcredit* OR micro‐credit* OR “micro enterprise*” OR microenterprise* OR micro‐enterprise* OR “micro entrepreneur*” OR microentrepreneur* OR micro‐entrepreneur* OR saving* OR micro‐saving* OR microsaving* OR “Smallholder financ*” OR “rural financ*” OR “rural credit” OR ROSCAs OR SHGs OR “group lending” OR “community savings” or “small loan*” or “small lend*” or ((bank or credit*) N3 cooperat*) or ((credit or loan* or lend*) N3 program*) or (community N3 (bank* or saving* or loan* or lend*)) or “income generat*” or grameen OR ROSCA* OR stokvel* OR ((financial OR economic) N2 (literacy OR education OR skills OR training OR knowledge OR capab*)) OR banking OR budgeting OR “money manag*” OR “consumption smoothing” OR rationing OR earmarking OR “bank account*” OR “youth account*” OR “lock box*” OR “piggy bank*” OR “saving box*” OR ((access* OR participat*) N3 (financ* OR credit OR saving* OR loan* OR lending)) OR “financial inclusion” OR “inclusive finance” OR fintech OR “mobile monies” OR M‐PESA OR “mobile banking” OR cashless)

133,480

S18 AB (micro‐finance OR “micro finance” OR microfinance OR micro‐loan* OR microloan* OR “micro loan*” OR microleas* OR micro‐leas* OR “micro leas*” OR microlending OR micro‐lending OR “micro lending” OR microinsurance OR micro‐insurance OR “micro insurance” OR “microgroup lending” OR microfranchis* OR micro‐franchis* OR “micro franchis*” OR “micro credit*” OR microcredit* OR micro‐credit* OR “micro enterprise*” OR microenterprise* OR micro‐enterprise* OR “micro entrepreneur*” OR microentrepreneur* OR micro‐entrepreneur* OR saving* OR micro‐saving* OR microsaving* OR “Smallholder financ*” OR “rural financ*” OR “rural credit” OR SHGs OR “group lending” OR “community savings” or “small loan*” or “small lend*” or ((bank or credit*) N3 cooperat*) or ((credit or loan* or lend*) N3 program*) or (community N3 (bank* or saving* or loan* or lend*)) or “income generat*” or grameen OR ROSCA* OR stokvel* OR ((financial OR economic) N2 (literacy OR education OR skills OR training OR knowledge OR capab*)) OR banking OR budgeting OR “money manag*” OR “consumption smoothing” OR rationing OR earmarking OR “bank account*” OR “youth account*” OR “lock box*” OR “piggy bank*” OR “saving box*” OR ((access* OR participat*) N3 (financ* OR credit OR saving* OR loan* OR lending)) OR “financial inclusion” OR “inclusive finance” OR fintech OR “mobile monies” OR M‐PESA OR “mobile banking” OR cashless)

155,180

S17 TI (micro‐finance OR “micro finance” OR microfinance OR micro‐loan* OR microloan* OR “micro loan*” OR microleas* OR micro‐leas* OR “micro leas*” OR microlending OR micro‐lending OR “micro lending” OR microinsurance OR micro‐insurance OR “micro insurance” OR “microgroup lending” OR microfranchis* OR micro‐franchis* OR “micro franchis*” OR “micro credit*” OR microcredit* OR micro‐credit* OR “micro enterprise*” OR microenterprise* OR micro‐enterprise* OR “micro entrepreneur*” OR microentrepreneur* OR micro‐entrepreneur* OR saving* OR micro‐saving* OR microsaving* OR “Smallholder financ*” OR “rural financ*” OR “rural credit” OR SHGs OR “group lending” OR “community savings” or “small loan*” or “small lend*” or ((bank or credit*) N3 cooperat*) or ((credit or loan* or lend*) N3 program*) or (community N3 (bank* or saving* or loan* or lend*)) or “income generat*” or grameen OR ROSCA* OR stokvel* OR ((financial OR economic) N2 (literacy OR education OR skills OR training OR knowledge OR capab*)) OR banking OR budgeting OR “money manag*” OR “consumption smoothing” OR rationing OR earmarking OR “bank account*” OR “youth account*” OR “lock box*” OR “piggy bank*” OR “saving box*” OR ((access* OR participat*) N3 (financ* OR credit OR saving* OR loan* OR lending)) OR “financial inclusion” OR “inclusive finance” OR fintech OR “mobile monies” OR M‐PESA OR “mobile banking” OR cashless)

36,831

S16 S12 or S13 or S14 or S15

30,115

S15 TI ((“literature search” OR “database search” OR “bibliographic* search” OR “comprehensive search” OR “extensive search” OR “exhaustive search” OR “purposive search” OR “representative search” or “systemat* search”)) OR AB ((“literature search” OR “database search” OR “bibliographic* search” OR “comprehensive search” OR “extensive search” OR “exhaustive search” OR “purposive search” OR “representative search” or “systemat* search”)) OR SU ((“literature search” OR “database search” OR “bibliographic* search” OR “comprehensive search” OR “extensive search” OR “exhaustive search” OR “purposive search” OR “representative search” or “systemat* search”))

863

S14 TI ((review N3 (effectiveness OR effects OR systemat* OR synth* OR integrat* OR map* OR methodologic* OR quantitative OR evidence OR literature))) OR AB ((review N3 (effectiveness OR effects OR systemat* OR synth* OR integrat* OR map* OR methodologic* OR quantitative OR evidence OR literature))) OR SU ((review N3 (effectiveness OR effects OR systemat* OR synth* OR integrat* OR map* OR methodologic* OR quantitative OR evidence OR literature)))

18,688

S13 TI ((“Meta regression” OR “meta synth*” OR “meta‐synth*” OR “meta analy*” OR “metaanaly*” OR “meta‐analy*” OR “metanaly*” OR “Metaregression” OR “Meta‐regression” OR “Methodologic* overview” OR “pool* analys*” OR “pool* data” OR “Quantitative* overview” OR “research integration”)) OR AB ((“Meta regression” OR “meta synth*” OR “meta‐synth*” OR “meta analy*” OR “metaanaly*” OR “meta‐analy*” OR “metanaly*” OR “Metaregression” OR “Meta‐regression” OR “Methodologic* overview” OR “pool* analys*” OR “pool* data” OR “Quantitative* overview” OR “research integration”)) OR SU ((“Meta regression” OR “meta synth*” OR “meta‐synth*” OR “meta analy*” OR “metaanaly*” OR “meta‐analy*” OR “metanaly*” OR “Metaregression” OR “Meta‐regression” OR “Methodologic* overview” OR “pool* analys*” OR “pool* data” OR “Quantitative* overview” OR “research integration”))

4,335

S12 TI (((Systematic* OR synthes*) N3 (Research OR evaluation* OR finding* OR thematic* OR report OR descriptive OR explanatory OR narrative OR meta* OR review* OR data OR literature OR studies OR evidence OR map OR quantitative OR study OR studies OR paper OR impact OR impacts OR effect* OR compar*))) OR AB (((Systematic* OR synthes*) N3 (Research OR evaluation* OR finding* OR thematic* OR report OR descriptive OR explanatory OR narrative OR meta* OR review* OR data OR literature OR studies OR evidence OR map OR quantitative OR study OR studies OR paper OR impact OR impacts OR effect* OR compar*))) OR SU (((Systematic* OR synthes*) N3 (Research OR evaluation* OR finding* OR thematic* OR report OR descriptive OR explanatory OR narrative OR meta* OR review* OR data OR literature OR studies OR evidence OR map OR quantitative OR study OR studies OR paper OR impact OR impacts OR effect* OR compar*)))

11,688

S11 S1 OR S2 OR S3 OR S4 OR S5 OR S6 OR S7 OR S8 OR S9 OR S10

1,066,885

S10 TI (Africa or Asia or Caribbean or “West Indies” or “South America” or “Latin America” or “Central America” OR “Middle East”) OR AB (Africa or Asia or Caribbean or “West Indies” or “South America” or “Latin America” or “Central America” OR “Middle East”) OR SU (Africa or Asia or Caribbean or “West Indies” or “South America” or “Latin America” or “Central America” OR “Middle East”) OR GE (Africa or Asia or Caribbean or “West Indies” or “South America” or “Latin America” or “Central America” OR “Middle East”)

156,848

S9 TI ((“transitional country” or “transitional countries”)) OR AB ((“transitional country” or “transitional countries”)) OR SU ((“transitional country” or “transitional countries”))

88

S8 TI ((lmic or lmics or “third world” or “lami country” or “lami countries”)) OR AB ((lmic or lmics or “third world” or “lami country” or “lami countries”)) OR SU ((lmic or lmics or “third world” or “lami country” or “lami countries”))

570

S7 TI (low N3 middle N3 countr*) OR AB (low N3 middle N3 countr*) OR SU (low N3 middle N3 countr*)

758

S6 TI (low* N1 (gdp or gnp or “gross domestic” or “gross national”)) OR AB (low* N1 (gdp or gnp or “gross domestic” or “gross national”)) OR SU (low* N1 (gdp or gnp or “gross domestic” or “gross national”))

237

S5 TI ((developing or less* N1 developed or “under developed” or underdeveloped or “middle income” or low* N1 income) N1 (economy or economies)) OR AB ((developing or less* N1 developed or “under developed” or underdeveloped or “middle income” or low* N1 income) N1 (economy or economies)) OR SU ((developing or less* N1 developed or “under developed” or underdeveloped or “middle income” or low* N1 income) N1 (economy or economies))

2,346

S4 TI ((developing or less* N1 developed or “under developed” or underdeveloped or “middle income” or low* N1 income or underserved or “under served” or deprived or poor*) N1 (countr* or nation* or population* or world)) OR AB ((developing or less* N1 developed or “under developed” or underdeveloped or “middle income” or low* N1 income or underserved or “under served” or deprived or poor*) N1 (countr* or nation* or population* or world)) OR SU ((developing or less* N1 developed or “under developed” or underdeveloped or “middle income” or low* N1 income or underserved or “under served” or deprived or poor*) N1 (countr* or nation* or population* or world))

22,624

S3 AB Afghanistan OR Albania OR Algeria OR Angola OR Antigua OR Barbuda OR Argentina OR Armenia OR Armenian OR Aruba OR Azerbaijan OR Bahrain OR Bangladesh OR Barbados OR Benin OR Belize OR Bhutan OR Bolivia OR Botswana OR Brazil OR Brasil OR “Burkina Faso” OR “Burkina Fasso” OR “Upper Volta” OR Burundi OR Urundi OR Cambodia OR “Khmer Republic” OR Kampuchea OR Cameroon OR Cameroons OR Cameron OR Camerons OR “Cape Verde” OR “Central African Republic” OR Chad OR Chile OR China OR Colombia OR Comoros OR “Comoro Islands” OR Comores OR Mayotte OR Congo OR Zaire OR “Costa Rica” OR “Cote d'Ivoire” OR “Ivory Coast” OR Cuba OR “Djibouti” OR “French Somaliland” OR Dominica OR “Dominican Republic” OR “East Timor” OR “East Timur” OR “Timor Leste” OR Ecuador OR Egypt OR “United Arab Republic” OR “El Salvador” OR Eritrea OR Ethiopia OR Fiji OR Gabon OR “Gabonese Republic” OR Gambia OR Gaza OR “Georgia Republic” OR “Georgian Republic” OR Ghana OR “Gold Coast” OR Grenada OR Guatemala OR Guinea OR Guam OR Guiana OR Guyana OR Haiti OR Honduras OR India OR Maldives OR Indonesia OR Iran OR Iraq OR Jamaica OR Jordan OR Kazakhstan OR Kazakh OR Kenya OR Kiribati OR Korea OR Kosovo OR Kyrgyzstan OR Kirghizia OR “Kyrgyz Republic” OR Kirghiz OR Kirgizstan OR “Lao PDR” OR Laos OR Lebanon OR Lesotho OR Basutoland OR Liberia OR Libya OR Madagascar OR “Malagasy Republic” OR Malaysia OR Malaya OR Malay OR Sabah OR Sarawak OR Malawi OR Nyasaland OR Mali OR “Marshall Islands” OR Mauritania OR Mauritius OR “Agalega Islands” OR Mexico OR Micronesia OR “Middle East” OR Moldova OR Moldovia OR Moldovian OR Mongolia OR Montenegro OR Morocco OR Ifni OR Mozambique OR Myanmar OR Myanma OR Burma OR Namibia OR Nepal OR Antilles OR “New Caledonia” OR Nicaragua OR Niger OR Nigeria OR “Mariana Islands” OR Oman OR Muscat OR Pakistan OR Palau OR Palestine OR Panama OR Paraguay OR Peru OR Philippines OR Philipines OR Phillipines OR Phillippines OR “Puerto Rico” OR Rwanda OR Ruanda OR “Saint Kitts” OR “St Kitts” OR Nevis OR “Saint Lucia” OR “St Lucia” OR “Saint Vincent” OR “St Vincent” OR “Grenadines” OR “Samoa” OR “Samoan Islands” OR “Navigator Island” OR “Navigator Islands” OR “Sao Tome” OR “Saudi Arabia” OR Senegal OR Seychelles OR “Sierra Leone” OR “Sri Lanka” OR “Solomon Islands” OR Somalia OR Sudan OR Suriname OR Surinam OR Swaziland OR Syria OR Tajikistan OR Tadzhikistan OR Tadjikistan OR Tadzhik OR Tanzania OR Thailand OR Togo OR “Togolese Republic” OR Tonga OR Trinidad OR Tobago OR Tunisia OR Turkey OR Turkmenistan OR Turkmen OR Uganda OR Ukraine OR Uruguay OR Uzbekistan OR Uzbek OR Vanuatu OR “New Hebrides” OR Venezuela OR Vietnam OR “Viet Nam” OR “West Bank” OR Yemen OR Zambia OR Zimbabwe OR Jamahiriya OR Jamahiryria OR Libia OR Mocambique OR Principe OR Syrian OR “Indian Ocean” OR Melanesia OR “Western Sahara”

973,710

S2 TI Afghanistan OR Albania OR Algeria OR Angola OR Antigua OR Barbuda OR Argentina OR Armenia OR Armenian OR Aruba OR Azerbaijan OR Bahrain OR Bangladesh OR Barbados OR Benin OR Belize OR Bhutan OR Bolivia OR Botswana OR Brazil OR Brasil OR “Burkina Faso” OR “Burkina Fasso” OR “Upper Volta” OR Burundi OR Urundi OR Cambodia OR “Khmer Republic” OR Kampuchea OR Cameroon OR Cameroons OR Cameron OR Camerons OR “Cape Verde” OR “Central African Republic” OR Chad OR Chile OR China OR Colombia OR Comoros OR “Comoro Islands” OR Comores OR Mayotte OR Congo OR Zaire OR “Costa Rica” OR “Cote d'Ivoire” OR “Ivory Coast” OR Cuba OR “Djibouti” OR “French Somaliland” OR Dominica OR “Dominican Republic” OR “East Timor” OR “East Timur” OR “Timor Leste” OR Ecuador OR Egypt OR “United Arab Republic” OR “El Salvador” OR Eritrea OR Ethiopia OR Fiji OR Gabon OR “Gabonese Republic” OR Gambia OR Gaza OR “Georgia Republic” OR “Georgian Republic” OR Ghana OR “Gold Coast” OR Grenada OR Guatemala OR Guinea OR Guam OR Guiana OR Guyana OR Haiti OR Honduras OR India OR Maldives OR Indonesia OR Iran OR Iraq OR Jamaica OR Jordan OR Kazakhstan OR Kazakh OR Kenya OR Kiribati OR Korea OR Kosovo OR Kyrgyzstan OR Kirghizia OR “Kyrgyz Republic” OR Kirghiz OR Kirgizstan OR “Lao PDR” OR Laos OR Lebanon OR Lesotho OR Basutoland OR Liberia OR Libya OR Madagascar OR “Malagasy Republic” OR Malaysia OR Malaya OR Malay OR Sabah OR Sarawak OR Malawi OR Nyasaland OR Mali OR “Marshall Islands” OR Mauritania OR Mauritius OR “Agalega Islands” OR Mexico OR Micronesia OR “Middle East” OR Moldova OR Moldovia OR Moldovian OR Mongolia OR Montenegro OR Morocco OR Ifni OR Mozambique OR Myanmar OR Myanma OR Burma OR Namibia OR Nepal OR Antilles OR “New Caledonia” OR Nicaragua OR Niger OR Nigeria OR “Mariana Islands” OR Oman OR Muscat OR Pakistan OR Palau OR Palestine OR Panama OR Paraguay OR Peru OR Philippines OR Philipines OR Phillipines OR Phillippines OR “Puerto Rico” OR Rwanda OR Ruanda OR “Saint Kitts” OR “St Kitts” OR Nevis OR “Saint Lucia” OR “St Lucia” OR “Saint Vincent” OR “St Vincent” OR “Grenadines” OR “Samoa” OR “Samoan Islands” OR “Navigator Island” OR “Navigator Islands” OR “Sao Tome” OR “Saudi Arabia” OR Senegal OR Seychelles OR “Sierra Leone” OR “Sri Lanka” OR “Solomon Islands” OR Somalia OR Sudan OR Suriname OR Surinam OR Swaziland OR Syria OR Tajikistan OR Tadzhikistan OR Tadjikistan OR Tadzhik OR Tanzania OR Thailand OR Togo OR “Togolese Republic” OR Tonga OR Trinidad OR Tobago OR Tunisia OR Turkey OR Turkmenistan OR Turkmen OR Uganda OR Ukraine OR Uruguay OR Uzbekistan OR Uzbek OR Vanuatu OR “New Hebrides” OR Venezuela OR Vietnam OR “Viet Nam” OR “West Bank” OR Yemen OR Zambia OR Zimbabwe OR Jamahiriya OR Jamahiryria OR Libia OR Mocambique OR Principe OR Syrian OR “Indian Ocean” OR Melanesia OR “Western Sahara”

971,403

S1 SU Afghanistan OR Albania OR Algeria OR Angola OR Antigua OR Barbuda OR Argentina OR Armenia OR Armenian OR Aruba OR Azerbaijan OR Bahrain OR Bangladesh OR Barbados OR Benin OR Belize OR Bhutan OR Bolivia OR Botswana OR Brazil OR Brasil OR “Burkina Faso” OR “Burkina Fasso” OR “Upper Volta” OR Burundi OR Urundi OR Cambodia OR “Khmer Republic” OR Kampuchea OR Cameroon OR Cameroons OR Cameron OR Camerons OR “Cape Verde” OR “Central African Republic” OR Chad OR Chile OR China OR Colombia OR Comoros OR “Comoro Islands” OR Comores OR Mayotte OR Congo OR Zaire OR “Costa Rica” OR “Cote d'Ivoire” OR “Ivory Coast” OR Cuba OR “Djibouti” OR “French Somaliland” OR Dominica OR “Dominican Republic” OR “East Timor” OR “East Timur” OR “Timor Leste” OR Ecuador OR Egypt OR “United Arab Republic” OR “El Salvador” OR Eritrea OR Ethiopia OR Fiji OR Gabon OR “Gabonese Republic” OR Gambia OR Gaza OR “Georgia Republic” OR “Georgian Republic” OR Ghana OR “Gold Coast” OR Grenada OR Guatemala OR Guinea OR Guam OR Guiana OR Guyana OR Haiti OR Honduras OR India OR Maldives OR Indonesia OR Iran OR Iraq OR Jamaica OR Jordan OR Kazakhstan OR Kazakh OR Kenya OR Kiribati OR Korea OR Kosovo OR Kyrgyzstan OR Kirghizia OR “Kyrgyz Republic” OR Kirghiz OR Kirgizstan OR “Lao PDR” OR Laos OR Lebanon OR Lesotho OR Basutoland OR Liberia OR Libya OR Madagascar OR “Malagasy Republic” OR Malaysia OR Malaya OR Malay OR Sabah OR Sarawak OR Malawi OR Nyasaland OR Mali OR “Marshall Islands” OR Mauritania OR Mauritius OR “Agalega Islands” OR Mexico OR Micronesia OR “Middle East” OR Moldova OR Moldovia OR Moldovian OR Mongolia OR Montenegro OR Morocco OR Ifni OR Mozambique OR Myanmar OR Myanma OR Burma OR Namibia OR Nepal OR Antilles OR “New Caledonia” OR Nicaragua OR Niger OR Nigeria OR “Mariana Islands” OR Oman OR Muscat OR Pakistan OR Palau OR Palestine OR Panama OR Paraguay OR Peru OR Philippines OR Philipines OR Phillipines OR Phillippines OR “Puerto Rico” OR Rwanda OR Ruanda OR “Saint Kitts” OR “St Kitts” OR Nevis OR “Saint Lucia” OR “St Lucia” OR “Saint Vincent” OR “St Vincent” OR “Grenadines” OR “Samoa” OR “Samoan Islands” OR “Navigator Island” OR “Navigator Islands” OR “Sao Tome” OR “Saudi Arabia” OR Senegal OR Seychelles OR “Sierra Leone” OR “Sri Lanka” OR “Solomon Islands” OR Somalia OR Sudan OR Suriname OR Surinam OR Swaziland OR Syria OR Tajikistan OR Tadzhikistan OR Tadjikistan OR Tadzhik OR Tanzania OR Thailand OR Togo OR “Togolese Republic” OR Tonga OR Trinidad OR Tobago OR Tunisia OR Turkey OR Turkmenistan OR Turkmen OR Uganda OR Ukraine OR Uruguay OR Uzbekistan OR Uzbek OR Vanuatu OR “New Hebrides” OR Venezuela OR Vietnam OR “Viet Nam” OR “West Bank” OR Yemen OR Zambia OR Zimbabwe OR Jamahiriya OR Jamahiryria OR Libia OR Mocambique OR Principe OR Syrian OR “Indian Ocean” OR Melanesia OR “Western Sahara”

972,854

## Appendix 2 ‐ MECIR checklist

Campbell Standards for reviews and their applicability to overviews of reviews. Note: this table is directly adapted from the Campbell MEC2IER standards and Table 1, Appendix S1 from Hartling, L., Chisholm, A., Thomson, D., & Dryden, D. (2012). A descriptive analysis of overviews of reviews published between 2000 and 2011. *PLOS One*, 7(11), e49667.

**Table:**

**Item No.**^*^^†^	**Item name**	**Standard**	**Applicability to overviews of reviews**
Setting the research question(s) to inform the scope of the review			
1	Formulating review questions	Ensure that the review question and particularly the outcomes of interest, address issues that are important to stakeholders such as consumers, health professionals and policy makers.	Directly applicable
2	Pre‐defining objectives	Define in advance the objectives of the review, including participants, interventions, comparators and outcomes.	Directly applicable
3	Considering potential adverse effects	Consider any important potential adverse effects of the intervention(s) and ensure that they are addressed.	Applicable. Overview authors should identify important outcomes including adverse effects and comment if any are not addressed or reported in the included SRs. If not addressed or reported in the SRs, overview authors need to decide whether to examine the primary studies to see if relevant outcomes were reported at the primary study level but not extracted at the SR level.
Setting eligibility criteria for including studies in the review			
5	Pre‐defining unambiguous criteria for participants	Define in advance the eligibility criteria for participants in the studies.	Directly applicable
7	Pre‐defining unambiguous criteria for interventions and comparators	Define in advance the eligible interventions and the interventions against which these can be compared in the included studies.	Directly applicable
8	Clarifying role of outcomes	Clarify in advance whether outcomes listed under ‘Criteria for considering studies for this review’ are used as criteria for including studies (rather than as a list of the outcomes of interest within whichever studies are included)	Directly applicable
9	Pre‐defining study designs	Define in advance the eligibility criteria for study designs in a clear and unambiguous way, with a focus on features of a study's design rather than design labels.	Directly applicable; need to define what is considered a SR.
12	Excluding studies based on publication status	Include studies irrespective of their publication status, unless explicitly justified.	Directly applicable
13	Changing eligibility criteria	Justify any changes to eligibility criteria or outcomes studied. In particular, post hoc decisions about inclusion or exclusion of studies should keep faith with the objectives of the review rather than with arbitrary rules.	Directly applicable
14	Pre‐defining outcomes	Define in advance which outcomes are primary outcomes and which are secondary outcomes.	Directly applicable
Planning the review methods at protocol stage			
19	Planning the search	Plan in advance the methods to be used for identifying studies. Design searches to capture as many studies as possible meeting the eligibility criteria, ensuring that relevant time periods and sources are covered and not restricting by language or publication status.	Directly applicable
20	Planning the assessment of risk of bias in included studies	Plan in advance the methods to be used for assessing risk of bias in included studies, including the tool(s) to be used, how the tool(s) will be implemented, and the criteria used to assign studies, for example, to judgements of low risk, high risk and unclear risk of bias.	Applicable. Overview authors should determine whether they will extract risk of bias assessments from the included SRs or conduct risk of bias assessments on the primary studies themselves. Overview authors should determine how they will handle discrepancies in approaches to risk of bias assessments across SRs. Overview authors should determine whether and how they will assess methodological quality of the included SRs.
21	Planning the synthesis of results	Plan in advance the methods to be used to synthesize the results of the included studies, including whether a quantitative synthesis is planned, how heterogeneity will be assessed, choice of effect measure (e.g. odds ratio, risk ratio, risk difference or other for dichotomous outcomes), and methods for meta‐analysis (e.g. inverse variance or Mantel Haenszel, fixed‐effect or random effects model).	Applicable. Overview authors should determine how they will present the data from included SRs and whether they will re‐analyze data to provide consistency (e.g., choice of effect measure, method of analysis).
22	Planning subgroup analyses	Pre‐define potential effect modifiers (e.g. for subgroup analyses) at the protocol stage; restrict these in number; and provide rationale for each.	Applicable. Overview authors should specify subgroups of interest and determine whether they will conduct additional analyses if subgroups of interest are not examined or reported in the included SRs.
Searching for studies			
24	Searching key databases	Search the Cochrane Review Group's Specialized Register (internally, e.g. via the Cochrane Register of Studies, or externally via CENTRAL). Ensure that CENTRAL and MEDLINE (e.g. via PubMed) have been searched (either for the review or for the Review Group's Specialized Register).	Applicable. Overview authors should search The Cochrane Library (i.e., Cochrane Database of Systematic Reviews and Database of Abstracts of Reviews of Effectiveness) and may wish to consult relevant Cochrane Review Groups for a listing of reviews.
32	Structuring search strategies for bibliographic databases	Inform the structure of search strategies in bibliographic databases around the main concepts of the review, using appropriate elements from PICO and study design. In structuring the search, maximize sensitivity whilst striving for reasonable precision. Ensure correct use of the AND and OR operators.	Directly applicable
33	Developing search strategies for bibliographic databases	Identify appropriate controlled vocabulary (e.g. MeSH, Emtree, including ‘exploded’ terms) and free‐text terms (considering, for example, spelling variants, synonyms, acronyms, truncation and proximity operators).	Directly applicable
35	Restricting database searches	Justify the use of any restrictions in the search strategy on publication date, publication format or language.	Directly applicable
36	Documenting the search process	Document the search process in enough detail to ensure that it can be reported correctly in the review.	Directly applicable
37	Rerunning searches	Rerun or update searches for all relevant databases within 12 months before publication of the review or review update, and screen the results for potentially eligible studies.	Directly applicable
Selecting studies into the review			
39	Making inclusion decisions	Use (at least) two people working independently to determine whether each study meets the eligibility criteria, and define in advance the process for resolving disagreements.	Directly applicable
40	Excluding studies without useable data	Include studies in the review irrespective of whether measured outcome data are reported in a ‘usable’ way.	Directly applicable
41	Documenting decisions about records identified	Document the selection process in sufficient detail to complete a PRISMA flow chart and a table of ‘Characteristics of excluded studies’.	Directly applicable
42	Collating multiple reports	Collate multiple reports of the same study, so that each study rather than each report is the unit of interest in the review.	Directly applicable (e.g., SR published in Cochrane Library and peer‐reviewed journal; published and unpublished version of he same SR).
Collecting data from included studies			
43	Using data collection forms	Use a data collection form, which has been piloted.	Directly applicable
44	Describing studies	Collect characteristics of the included studies in sufficient detail to populate a table of ‘Characteristics of included studies’.	Directly applicable
46	Extracting outcome data in duplicate	Use (at least) two people working independently to extract outcome data from reports of each study, and define in advance the process for resolving disagreements.	Directly applicable
47	Making maximal use of data	Collect and utilize the most detailed numerical data that might facilitate similar analyses of included studies.	Applicable. Overview authors should extract detailed data from meta‐analyses when available that will facilitate comparisons across SRs.
50	Choosing intervention groups in multiarm studies	If a study is included with more than two intervention arms, include in the review only intervention and control groups that meet the eligibility criteria.	Overview authors should be aware of how SR authors have handled such studies.
51	Checking accuracy of numeric data in the review	Compare magnitude and direction of effects reported by studies with how they are presented in the review, taking account of legitimate differences.	Applicable. Caution is needed when comparing interventions that have not been formally compared in either direct or indirect analyses.
Assessing risk of bias in included studies			
52	Assessing risk of bias	Assess the risk of bias for each included study.	Determine a priori whether overview authors will assess the methodological quality of included SRs and what tool will be used.
53	Assessing risk of bias in duplicate	Use (at least) two people working independently to apply the risk of bias tool to each included study, and define in advance the process for resolving disagreements.	Applicable based on assessing methodological quality of SRs.
54	Supporting judgements of risk of bias	Justify judgements of risk of bias (high, low and unclear) and provide this information in the ‘Risk of bias’ tables (as ‘Support for judgement’).	Applicable based on assessing methodological quality of SRs.
61	Incorporating assessments of risk of bias	If randomized trials have been assessed using one or more tools in addition to the Cochrane ‘Risk of bias’ tool, use the Cochrane tool as the primary assessment of bias for interpreting results, choosing the primary analysis, and drawing conclusions.	Applicable to extracting and reporting risk of bias assessments for individual studies that were included in the included SRs.
Summarizing the findings			
76	Assessing the quality of the body of evidence	Use the five GRADE considerations (study limitations, consistency of effect, imprecision, indirectness and publication bias) to assess the quality of the body of evidence for each outcome, and to draw conclusions about the quality of evidence within the text of the review.	Extract quality of evidence assessments from the included SRs. Decide a priori what to do if quality of evidence assessments have not been performed or performed inconsistently across SRs.
77	Justifying assessments of the quality of the body of evidence	Justify and document all assessments of the quality of the body of evidence (for example downgrading or upgrading if using the GRADE tool).	Extract relevant information from the SRs.
Reaching conclusions			
78	Formulating implications for practice	Base conclusions only on findings from the synthesis (quantitative or narrative) of studies included in the review.	Directly applicable
79	Avoiding recommendations	Avoid providing recommendations for practice.	Directly applicable

* The items listed are among those considered mandatory for Cochrane Intervention Reviews. The item numbers, names, and standards are from: Chandler J, Churchill R, Higgins J, Lasserson T, Tovey D. Methodological standards for the conduct of new Cochrane Intervention Reviews. Version 2.1, 8 December 2011.

† The section from the above citation on “synthesizing the results of included studies” has been omitted from this table as it relates to the quantitative synthesis of individual studies in a meta‐analysis. For the most part, overviews of reviews have been descriptive in nature. Guidance on performing indirect analyses or mixed treatment comparisons is beyond the scope of this paper.
